# Amniotic MSC affect CD8 naive polarization toward SLEC/MPEC subsets by down-modulating IL-12Rβ1 and IL-2Rα signaling pathways

**DOI:** 10.1016/j.isci.2023.108483

**Published:** 2023-11-17

**Authors:** Andrea Papait, Elsa Vertua, Patrizia Bonassi Signoroni, Anna Cargnoni, Marta Magatti, Francesca Romana Stefani, Jacopo Romoli, Antonietta Rosa Silini, Ornella Parolini

**Affiliations:** 1 Department of Life Science and Public Health, Università Cattolica del Sacro Cuore, 00168 Rome, Italy; 2 Fondazione Policlinico Universitario “Agostino Gemelli” IRCCS, 00168 Rome, Italy; 3 Centro di Ricerca E. Menni, Fondazione Poliambulanza Istituto Ospedaliero, 25124 Brescia, Italy

**Keywords:** Immunology, Components of the immune system, Cell biology, Stem cells research

## Abstract

Mesenchymal stromal cells (MSCs) are known for their immunomodulatory activity. Here, we report that MSCs isolated from the amniotic membrane of human term placenta (hAMSCs) impact CD8 T cell fate through a multifaceted mechanism. We observed that hAMSCs are able to impact the metabolism of naive CD8 lymphocytes by downregulating the phosphorylation of mTOR and AKT, thus blocking cell differentiation. This effect is due to the ability of hAMSCs to reduce the expression of two receptors, IL-12Rβ1 and IL-2RA, resulting in reduced phosphorylation of STAT4 and STAT5. In addition, hAMSCs reduce the expression of two transcriptional factors, Tbet and Eomes, directly involved in early effector cell commitment. Our results unravel an unknown feature of MSCs, offering alternative mechanistic insights into the effects of MSCs for the treatment of diseases characterized by an altered activation of memory subsets, such as autoimmune diseases and graft versus host disease.

## Highlights

• HAMSCs have an impact on the early phase of CD8 T cell differentiation
• HAMSC impact on CD8 T cell metabolism (mTOR pathway)
• HAMSCs triggers changes in the transcriptional landscape of CD8 T cells
• HAMSCs affect the signaling cascade downstream the IL-12 and IL-2 receptor

## Graphical abstract

**Figure:**
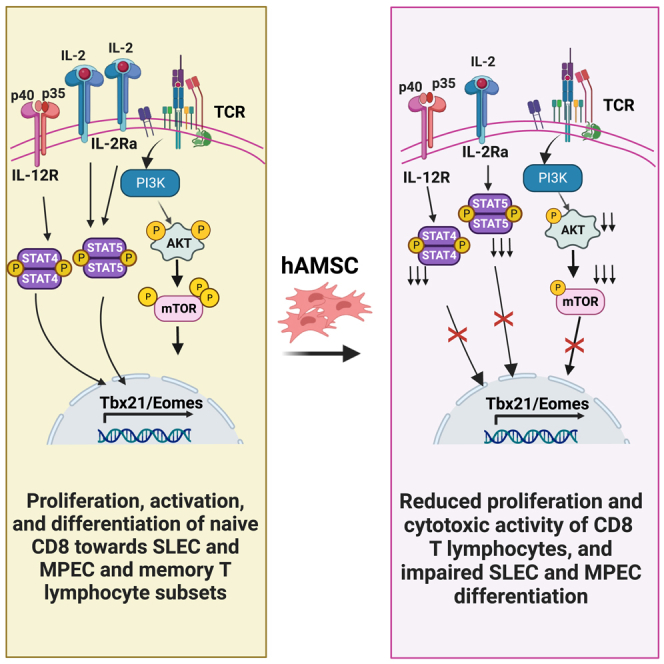

## Introduction

The formation of an adaptive T cell memory repertoire is a key feature of the primary adaptive immune response.

Shortly after activation, CD8 effector cells are differentiated into short-lived effector cells (SLECs), characterized by low survival and high proliferation potential, and long-lived memory precursor effector cells (MPECs) characterized by developing into memory cells.^1^^,^^2^

Antigen-specific memory CD8 T cells constitute the long-lived arm of immunity that rapidly protects the organism from secondary infections or tumors^3^ and the inability to establish proper CD8^−^driven immunological memory underlies numerous pathologies such as autoimmune diseases and allograft rejection^4^^,^^5^^,^^6^

Mesenchymal stromal cells (MSCs) are versatile stromal components whose known role in modulating immunity and inflammation has been exploited in numerous clinical trials ranging from their use in the treatment or prevention of graft versus host disease (GvHD) to the treatment of autoimmune diseases such as Crohn’s disease and neurodegenerative disorders.^7^^,^^8^ MSCs target a wide range of immune cells through cell-cycle arrest.^9^^,^^10^ One pivotal mechanism underlying this suppression involves interference with amino acid metabolism within the inflammatory microenvironment, in which the enzyme indoleamine 2,3-dioxygenase (IDO) has been shown to play a role.^11^ Other molecules with immunomodulatory properties that are secreted by MSCs include transforming growth factor-β1, hepatocyte growth factor,^12^ prostaglandin E2,^13^ and soluble human leukocyte antigen G (HLA-G).^14^ Notably, tumor necrosis factor-α (TNF-α)-stimulated gene 6 protein (TSG-6) has emerged as a recent addition to the repertoire of anti-inflammatory factors secreted by human MSCs.^15^

Recently, efferocytosis has been reported as another mechanism responsible for the immune modulatory actions of MSCs. Upon infusion, apoptotic MSCs undergo phagocytosis by recipient phagocytes, leading to their functional polarization toward inhibitory phenotypes, and to the production of indoleamine 2,3-dioxygenase, thus triggering the immunomodulatory effect.^16^

MSCs have been successfully identified and isolated from various tissues, encompassing not only adult sources such as bone marrow and adipose tissue, but also fetal sources such as the umbilical cord and placenta.^17^ This has propelled clinical research toward the use of different MSC subtypes due to their distinct characteristics, such as tissue availability, donor risk considerations, ease of isolation, which has led to a proliferation of clinical trials.^17^^,^^18^

Among the diverse types of MSCs, those derived from the amniotic membrane of the human term placenta (hAMSCs) have demonstrated distinct immunomodulatory properties. Indeed, unlike MSCs isolated from sources such as bone marrow, hAMSCs do not require priming to exert their immunomodulatory actions.^19^^,^^20^ In this context, hAMSCs have exhibited the capacity to influence the activation and differentiation of various components of adaptive immunity, including T cells,^21^^,^^22^ whereby they have been shown to block the polarization toward inflammatory Th subsets,^21^^,^^22^^,^^23^^,^^24^ and induce Treg regulatory cells.^22^^,^^25^ In addition, they have been shown to block the maturation and differentiation of monocytes into antigen-presenting cells (APCs) while promoting polarization and the acquisition of anti-inflammatory properties typical of M2 macrophages.^26^^,^^27^ Preclinical studies have shown that hAMSCs can exert therapeutic effects in animal models of acute and chronic inflammation-related diseases.^28^^,^^29^^,^^30^^,^^31^^,^^32^ Furthermore, it is now known that many of the immunoregulatory actions of hAMSCs occur via paracrine mechanisms, underscoring the clinical relevance of the factors contained and mediated in their secretome.^33^^,^^34^^,^^35^

Here, we investigated a topic not yet explored, namely the potential impact of MSCs and in particular of hAMSCs and their secreted factors on the maturation and formation of the memory CD8 lymphocyte repertoire.

In this study we focused on the effect of hAMSC on the early, post-activation stages of naive CD8 lymphocytes, where the expression of two pivotal transcriptional factors, Tbet and Eomes, drive the differentiation of naive toward SLECs and long-lived MPECs CD8 T cells, respectively.^36^^,^^37^ We demonstrated that hAMSCs modulate the expression of Tbet and Eomes leading to reduced polarization toward the MPEC compartment, thus suggesting the capacity of hAMSC to counteract the formation of memory CD8 T lymphocytes. This effect is related to the capacity of hAMSC to directly impact CD8 T lymphocyte activation by reducing the phosphorylation of AKT and mTOR. Finally, we show that hAMSC downregulate the expression of IL-2Rα and IL-12Rβ1 receptor resulting in reduced phosphorylation of STAT5 and STAT4, which are two key factors in governing the differentiation of CD8 naive lymphocytes.^38^

## Results

### hAMSCs harbor the potential to determine T cell fate

To investigate the impact of hAMSCs on immunological CD8 T cell memory commitment, we first analyzed the effect of hAMSCs on the activation and engagement of CD8 T lymphocytes within the peripheral blood mononuclear cell (PBMC) stimulated with anti CD3 mAbs over time.

**Figure 1:**
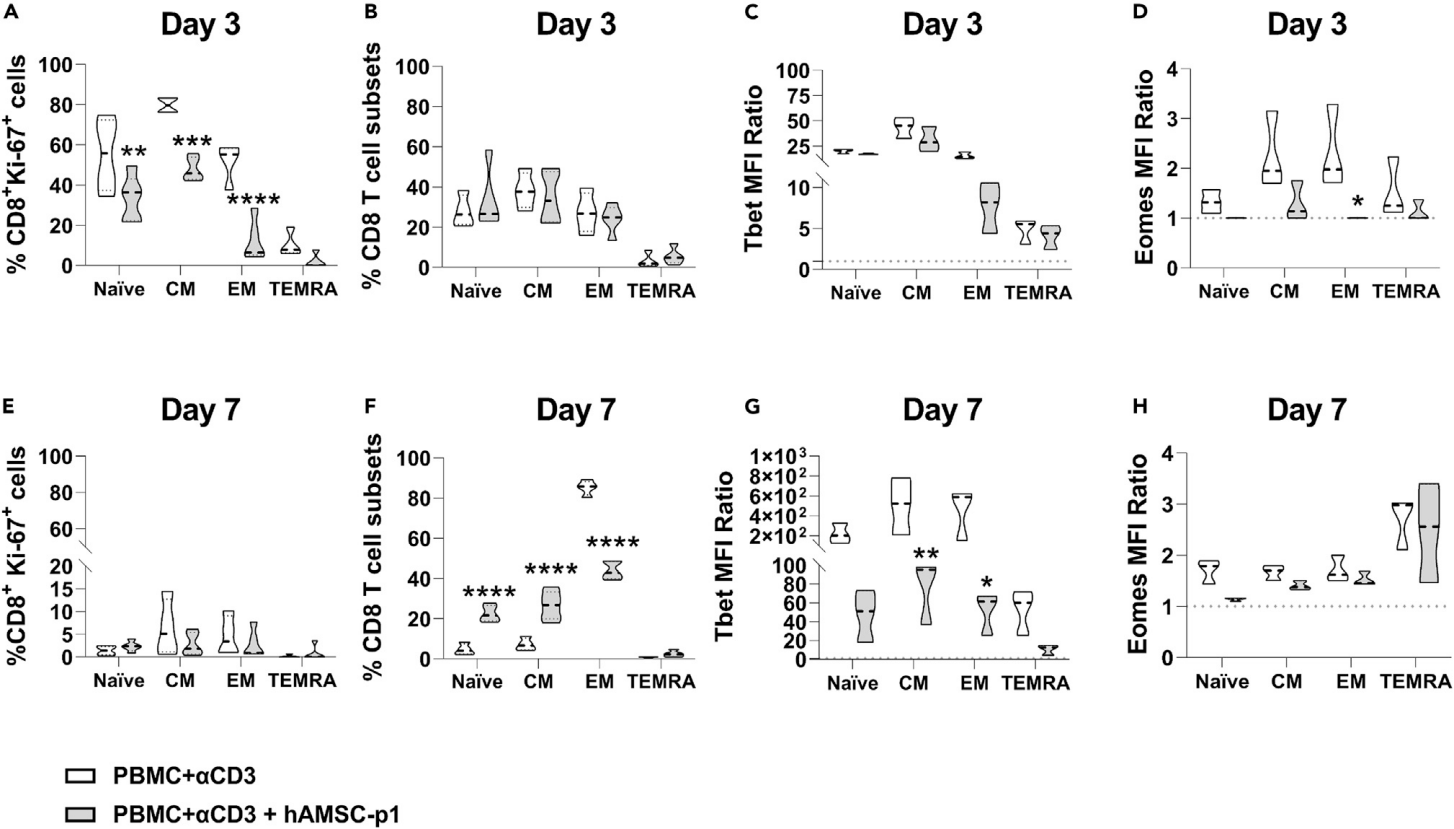
hAMSCs harbor the potential to determine T cell fate (A–H) PBMC stimulated with antiCD3 antibody were co-cultured with hAMSCs p1 (gray) for 3 (upper panels) or 7 (lower panels) days. Cell proliferation was evaluated as percentage of Ki67+ cells for the different CD8^+^ subsets investigated at day 3 (A) and 7 (E). Cells were then analyzed by flow cytometry to discriminate the CD8 naive T cell pool from the memory subsets as follows: naive T lymphocytes (CD197+CD45RO-), central memory (CM) (CD197+CD45RO+), effector memory (EM) (CD197−CD45RO+) and effector memory RA (TEMRA) (CD197-CD45RO-). CD8 T cell differentiation is represented as the percentage of the different subsets identified at day 3 (B) and 7 (F). The expression of transcription factors Tbet and Eomes within these subsets was quantified as mean fluorescence intensity (MFI) ratios at day 3 for Tbet (C) and Eomes (D) and at day 7 for Tbet (G) and Eomes (H). Results are displayed as violin plots showing median (dashed line), 25th and 75th quartiles (∗p < 0.05, ∗∗p < 0.01, ∗∗∗∗p < 0.001); stimulated PBMC alone represent the control group (white); N = 5 independent experiments performed starting from 5 PBMC donors and 6 different hAMSC preparation.

Co-culture of PBMCs with hAMSCs, in cell-to-cell contact, reduced proliferation of both naive and differentiated CD8 T cell subsets (central memory (CM), effector memory (EM), and EM RA (TEMRA) at day 3 (Figure 1A). This effect was no longer detectable at day 7, since, at which time, CD8 proliferation was drastically reduced and the eventual effect of hAMSCs on CD8 proliferation was difficult to assess (Figure 1E). To determine whether the effect on T cell proliferation reflected changes in CD8 T cell memory commitment, we analyzed the proportion of different memory subsets generated from naive cells after treatment with hAMSCs. At day 3, differentiation of naive cells in CM, EM, and TEMRA was not affected by hAMSCs conditioning (Figure 1B). Conversely, at day 7, the EM pool was reduced and its reduction was counterbalanced by the preservation of naive and CM pools (Figure 1F). Accordingly, the flow cytometry analysis indicated a drastic reduction in the expression of Tbet and Eomes transcription factors after co-culture with hAMSCs. These are two transcription factors involved in the early stages of CD8 lymphocyte commitment to memory subsets. Notably, Tbet reduction was evident by day 3 and the reduction became statistically significant at day 7, particularly in the polarization toward the CM and EM subsets (Figures 1C and 1G). The influence of hAMSCs on Eomes transcription factor expression was notably more significant on day 3, resulting in a noticeable reduction in all examined subsets, although statistical significance was achieved only for the EM subset. By day 7, differences between cells co-cultured with hAMSCs and the control group had largely diminished for all the investigated subsets (Figures 1D and 1H).

### hAMSCs directly orchestrate T lymphocyte commitment

To define the direct role of hAMSCs in CD8 T cell commitment, experiments were also performed with purified CD8 naive T lymphocytes stimulated with antiCD3 and antiCD28 mAbs in the presence of IL -12 and IL -2.^39^^,^^40^

**Figure 2:**
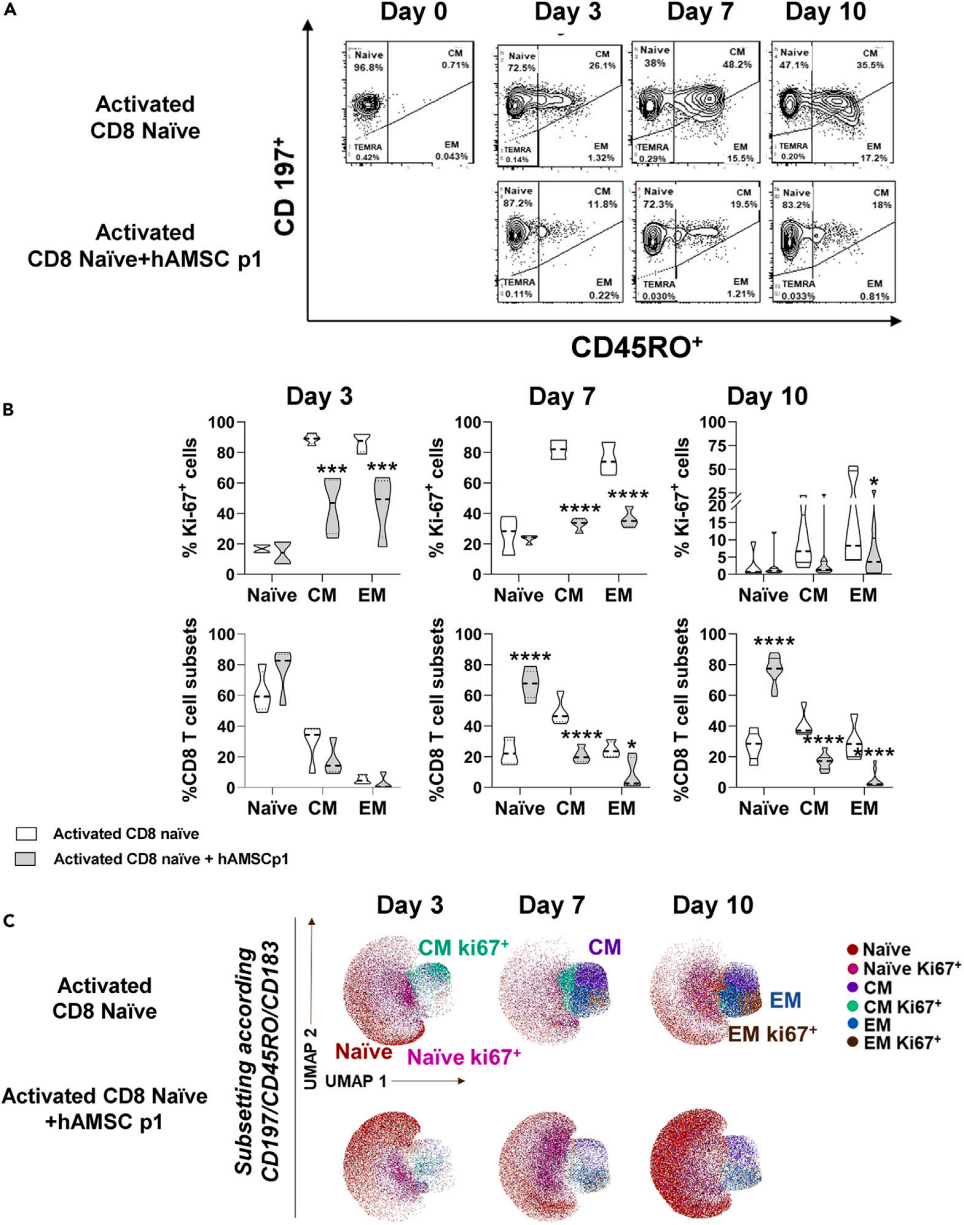
hAMSCs directly orchestrate T lymphocyte commitment Purified CD8 naive T lymphocytes were stimulated with antiCD3, antiCD28, and the exogeneous administration of IL-12 and IL-2, and cultured in the presence of hAMSCs (gray). (A and B) CD8 T cells were allowed to differentiate for 10 days and the degree of proliferation (as percentage of Ki67+ cells) and commitment (as percentage of the different T cell subsets) evaluated at day 3, 7, and 10 by flow cytometry. CD8 T cells were distinguished based on the differential expression of CD197 and CD45RO in naive (CD197+CD45RO-), central memory (CM) (CD197+CD45RO+) and effector memory (EM) (CD197−CD45RO+). (C) Uniform Manifold Approximation and Projection (UMAP) representation of the CD8^+^ T cell landscape obtained by Clusterexplorer plugin. Cells were stratified for CD197/CCR7, CD45RO and CD183 and subsequently clustered based on the expression or not of the proliferation marker Ki67, allowing to identify 6 clusters (Naive Ki67-, Naive Ki67+, CM Ki67-, CM Ki67+, EM Ki67-, and EM Ki67+ (C).Results in (A) and (B) are displayed as violin plots showing median (dashed line), 25th and 75th quartiles (∗p < 0.05, ∗∗p < 0.01, ∗∗∗∗p < 0.001); stimulated CD8 T cells alone represent the control group (white); N = 4 independent experiments performed starting from 4 purified CD8 naive donors and 4 different hAMSC preparation.

After activation, naive CD8 T cells progressively differentiate toward CM and EM subtypes (Figure 2A). Proliferation of naive, CM, and EM reached significant levels following activation, approximately three days after stimulation and peaks on day 7. Specifically, at day 7 approximately 80% of CMs and EMs proliferated (Figure 2B upper panel). When activation of naive CD8 T cells was performed in contact with hAMSC, the proliferation of naive T cells was not affected, instead the proliferation of both CM and EM was significantly reduced by hAMSC, throughout the period investigated (Figure 2B upper panel). Consequently, the commitment of cells to specific subpopulations was hindered, with a notable preservation of the naive cell fraction apparent at day 7. This was accompanied by a concomitant decrease in the polarization toward the CM and EM subpopulations (Figures 2A and 2B, lower panel). The observed effect, nonetheless, does not appear to be attributable to an increase in mortality (Figures S1A and S1B). To visualize the differentiation process over time and understand whether hAMSCs could induce the acquisition of distinctive features in CD8 T cells or differentiation into a different cellular subset, we conducted high-dimensional flow cytometry analysis, considering the expression of the activation marker CD183 and the proliferation marker Ki67. This approach has allowed for the identification of subclusters within different subpopulations that were actively proliferating. These subclusters indeed were characterized by their simultaneous association with a particular subset (Naive, CM, EM) and their positive expression of the proliferation marker Ki67. Once again, the impact of hAMSCs was evident in the reduction of the density of double-positive clusters, which was further reflected in a substantial accumulation of CD8 naive lymphocytes by day 10. These findings are in line with the results presented in Figure 2B, upper panel. This effect is lost in the control condition, in absence of hAMSCs, due to stimulus-induced differentiation.

### hAMSCs influence early phases of CD8 naive T cell commitment

To further evaluate hAMSCs as determinants of T cell fate, we analyzed their ability to drive the differentiation of naive CD8 T cells toward the SLEC and MPEC subtypes. These two subtypes can be distinguished by the differential expression of two surface markers, CD183 and KLRG1. SLECs are defined as CD183- KLRG1+, whereas MPECs are characterized by expression of CD183 and loss of positivity for KLRG1. We also examined the frequency of early effector cells (EECs), which function as master precursor effector T cells and are characterized by co-expression of the two markers mentioned previously (CD183+KLRG1+),^40^ and the frequency of double-negative effector cells (DNECs), whose role in CD8 lymphocyte commitment remains unclear.^40^

**Figure 3:**
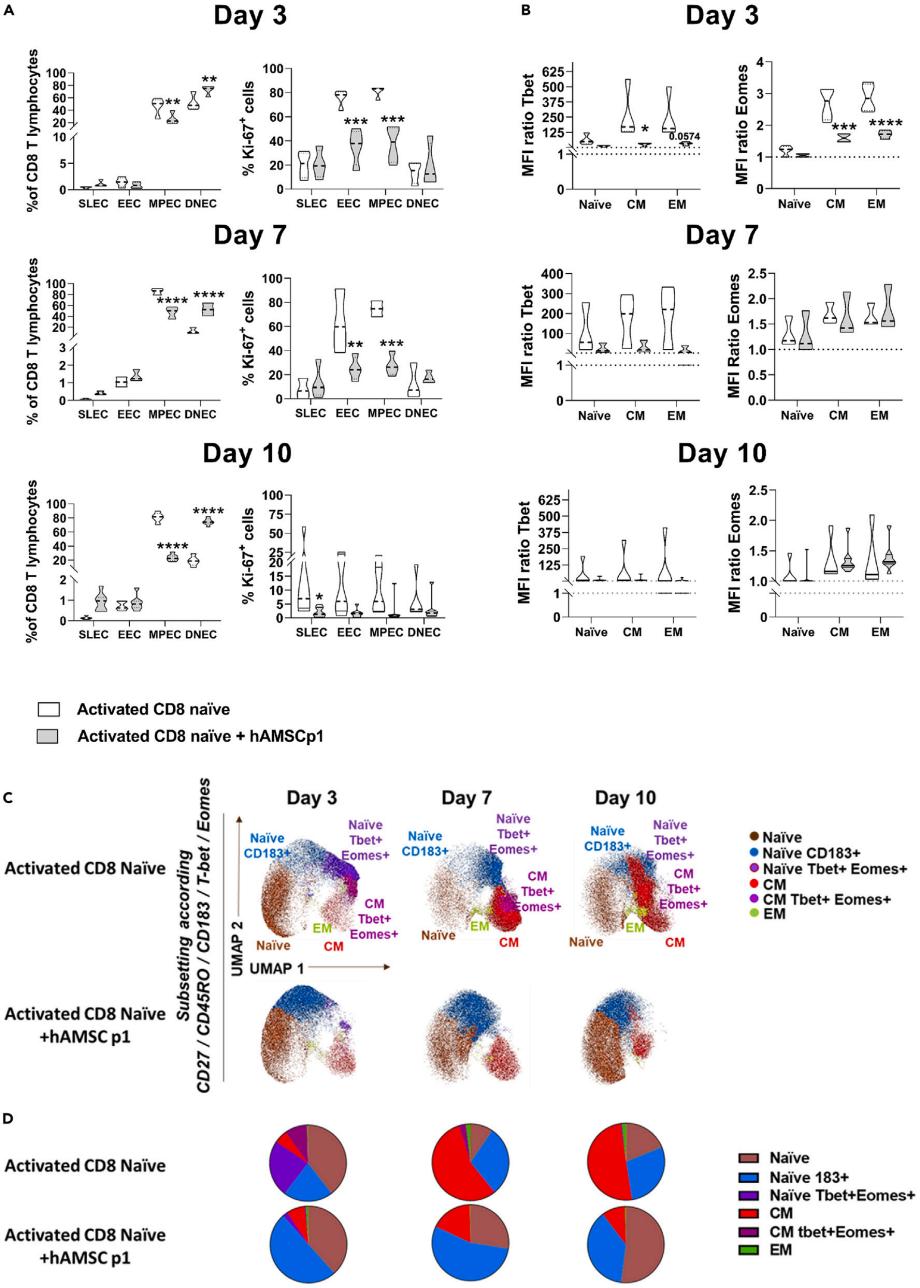
hAMSCs influence early phases of CD8 naive T cell commitment Purified CD8 naive T lymphocytes were stimulated with antiCD3, antiCD28, and the exogeneous administration of IL-12 and IL-2, and cultured in the presence of hAMSCs (gray). CD8 T cells were allowed to differentiate for 10 days and the different cellular subtypes analyzed at day 3, 7, and 10 as relative frequency (left panel 3A or as % of Ki67 proliferating cells). (A) CD3^+^CD8^+^ T lymphocytes were further analyzed for the expression of CD183 and KLRG1 and classified into four populations: SLECs (CD183- KLRG1+), EECs (CD183+ KLRG1+), MPECs (CD183+ KLRG1-), and DNECs (CD183- KLRG1-). (B) The expression of the transcription factors Tbet and Eomes was evaluated at all time points. (C) Uniform Manifold Approximation and Projection (UMAP) representation of the CD8 T cell landscape obtained by Clusterexplorer plugin. (D) Pie charts representing the distribution levels of the 6 identified clusters as a percentage of the total CD8 pool. Results in (A) and (B) are displayed as violin plots showing median (dashed line), 25th and 75th quartiles (∗p < 0.05, ∗∗p < 0.01, ∗∗∗∗p < 0.001); activated CD8 T cells alone represent the control group (white); N = 3 independent experiments performed starting from 3 purified CD8 naive donors and 4 different hAMSC preparation.

Having confirmed the ability of activated naive CD8 T lymphocytes to differentiate into MPECs in the presence of exogenous IL-12 and IL-2 (Figure 3A),^39^ we next examined the effects of hAMSCs on this early commitment phase. As shown in Figure 3A, treatment with hAMSCs resulted in a reduction in the MPEC compartment in terms of both relative frequency (Figure 3A left panel) and absolute number (Figure S2). In parallel, in presence of hAMSC the percentage of DNEC fraction increased (Figure 3A left panel), whereas no difference was observed in the absolute number of DNEC T cells (Figure S2). From the proliferation analysis of the different subsets, we observed that co-culture with hAMSCs leads to a significant reduction in the EEC and MPEC subsets at day 3 (Figure 3A, right panel). This difference persisted even on day 7 (Figure 3A, right panel). The transcription factors Tbet and Eomes promote the differentiation of naive CD8 lymphocytes.^37^ Here, we observed a substantial reduction in the mean fluorescence intensity (MFI) ratio of Tbet and Eomes on both CM and EM at day 3 from challenge with hAMSCs, a reduction that persisted until day 10, although not significantly (Figure 3B).

We next sought to identify the appearance of different CD8 T cell subsets or clusters over time. The unsupervised analysis was performed as aforementioned and shown in Figure 3C. Co-culture with hAMSCs allowed to retain a large amount of naive T lymphocytes (in brown) and increased the proportion of CD183 naive T lymphocytes (blue), suggesting that hAMSCs do not affect the early stages of activation of naive CD8 lymphocytes. However, hAMSCs strongly reduced the percentage of naive T lymphocytes positive for the expression of Tbet and Eomes (purple) as well as the commitment toward the CM (red and dark purple) and EM (green) memory subsets (Figure 3D).

### hAMSCs affect CD8 T cell metabolism

**Figure 4:**
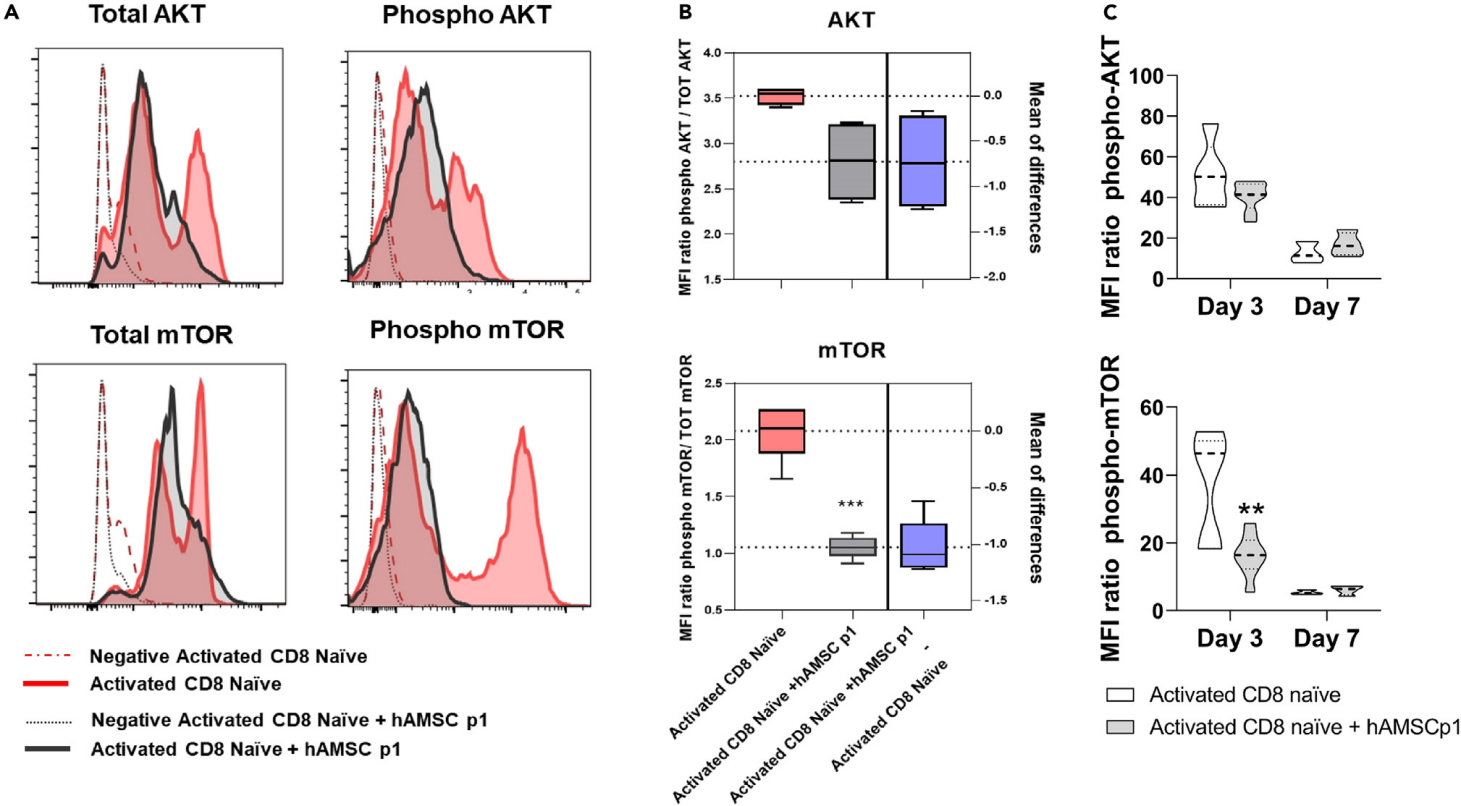
hAMSCs affect CD8 T cell metabolism Purified CD8 naive T lymphocytes were stimulated with antiCD3, antiCD28, and the exogeneous administration of IL-12 and IL-2, and cultured in the presence of hAMSCs. Cells were harvested at day 3 and analyzed by flow cytometry. Specifically, CD8 T cells were stained for phospho mTOR, phospho AKT, and for their total protein content. (A and B) Representative plots of the MFI ratio between total protein and relative phosphorylated protein. (C) Comparison of AKT and mTOR phosphorylation at the two different time points, 3 and 7 days. Results in (B) are displayed estimation plot with Tukey variation. Results in (C) are displayed as violin plots showing median (dashed line), 25th and 75th quartiles ∗∗∗p < 0.001; activated CD8 T cells alone represent the control group; N = 3 independent experiments performed starting from 3 purified CD8 naive donors and 4 different hAMSC preparation.

The PI3K/mTOR pathway is involved in the activation, proliferation, and differentiation of CD8 T lymphocytes.^41^ Because mTOR and its two main complexes, mTORC1 and mTORC2, control the differential expression of Tbet and Eomes,^42^^,^^43^ we next wanted to investigate the effect of hAMSCs on these important regulators of cellular metabolism. After three days of co-culture with hAMSCs, the total and phosphorylated forms of AKT and mTOR were reduced in activated CD8 T lymphocytes (Figure 4A). These observations suggest that hAMSCs may negatively affect the metabolic switch of naive CD8 T lymphocytes from fatty acid to glycolytic metabolism, which is required for full maturation of CD8 T lymphocytes.^44^

Representative estimation plots of the MFI ratio between total protein and relative phosphorylated protein demonstrate that hAMSCs reduce the phosphorylation of AKT, leading to a substantial reduction in mTOR phosphorylation and activation (Figure 4B).

When we compared the changes in the phosphorylation status of AKT and mTOR on day 7, we observed a drastic reduction in the phosphorylation of these two proteins, very likely because the commitment process is an early event, and therefore appreciable on day 3, and not on day 7 where the commitment process has already occurred (Figure 4C). This suggests a potential mechanism in which hAMSCs function to counteract the activation and differentiation of naive CD8 lymphocytes during the early days of commitment by inhibiting AKT and mTOR signaling.

### hAMSCs modulate the transcriptional landscape of naive CD8 T lymphocytes

To outline the transcriptional landscape peculiar to the commitment of naive CD8 T lymphocytes upon hAMSCs stimuli, we performed a high throughput gene expression analysis focusing on several genes involved in differentiation and metabolic processes, as well as epigenetic and transcriptional regulation of naive CD8 lymphocytes. We performed an unsupervised hierarchical clustering analysis and identified genes whose expression is over 2-fold higher and statistically significant (p < 0.05) in the early (24 h) post-activation stages (Figure S3).

**Figure 5:**
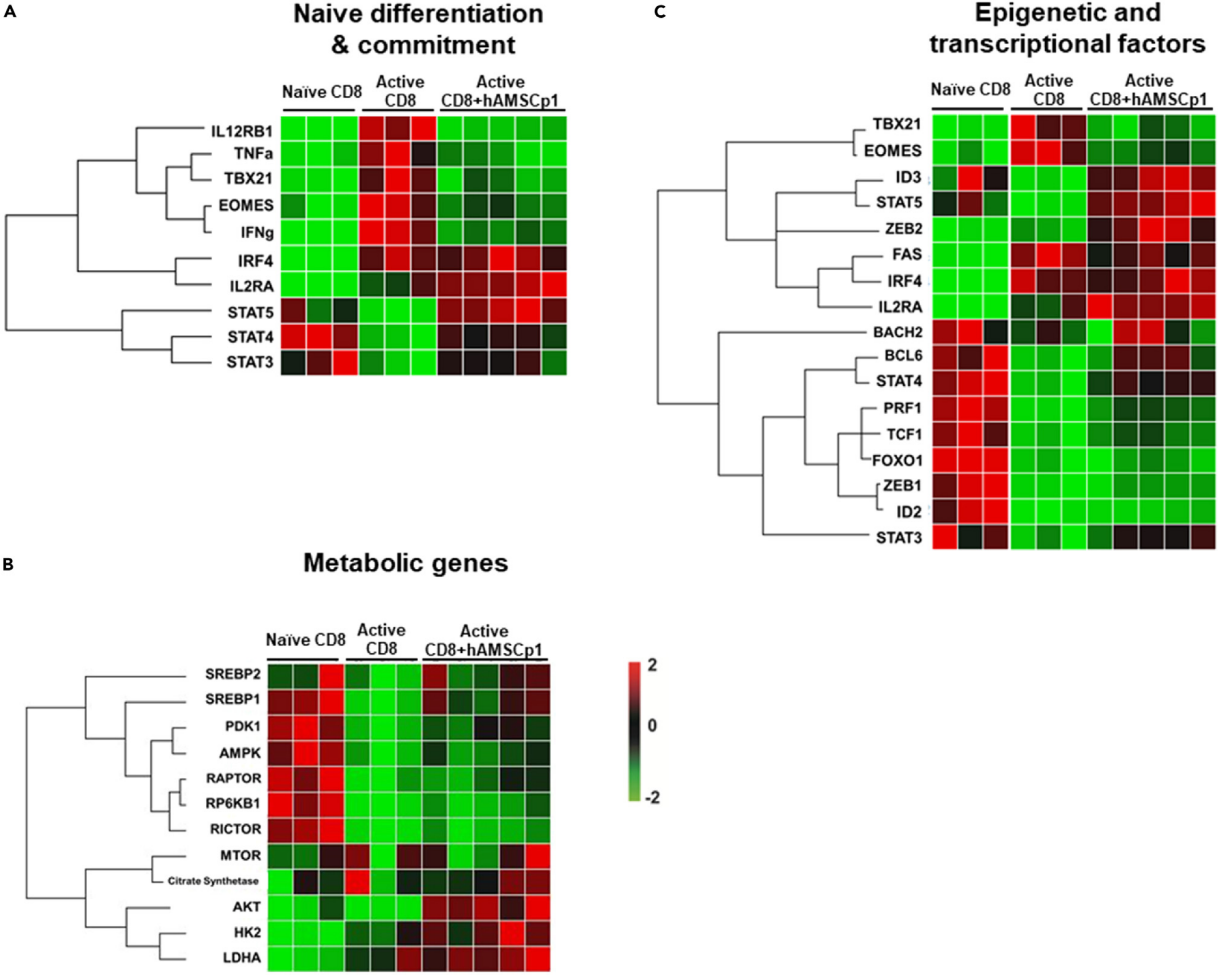
hAMSCs modulate the transcriptional landscape of naive CD8 T lymphocytes Transcriptomic profile of naive, activated and activated in the presence of hAMSCs CD8 T cells 24 h upon activation. Panels show metabolic related genes (A), epigenetic and transcriptional factors (B), and genes strictly related to naive CD8 T cell differentiation (C). The mRNA expression profile is represented as heatmap graph. The genes were clustered according to their expression patterns. N = 3 independent experiments performed starting from 3 purified CD8 naive donors and 5 different hAMSC preparation.

Having confirmed the upregulation of genes such as IL-12 receptor beta 1 (*IL12RB1*) and interleukin-2 receptor alpha (*IL2RA*), involved in the early stages of CD8 T cell activation, we next interrogated on determinants of cell fate. As shown in Figure 5A and Figure S3, the two main inflammatory cytokines, *IFNG* and *TNF*, typically induced in the early differentiation steps, were upregulated in activated cells.^38^^,^^45^ At the same time, also *IRF4* and the two master regulators of SLEC and MPEC commitment, *TBX21* (Tbet) and *EOMES* (Eomesodermin), respectively, were upregulated. Conversely, the expression of STAT genes (*STAT3, 4* and *5*), modulators of both activation and commitment of CD8 T lymphocytes, was higher in naive CD8 T cells compared to activated cells. Interestingly, when naive CD8 T lymphocytes were activated in the presence of hAMSCs, their gene expression profile was comparable to that of unstimulated naive CD8 T lymphocytes, with the exception of *IL2RA* and *IRF4*, whose increase upon activation was maintained also in the presence of hAMSCs.

Next, we focused our attention on genes involved in metabolic switching. As shown in Figures 5B and S3, *SREBP1* and *2*, *AMPK*, *PDK1*, *RAPTOR*, and *RICTOR* resulted downregulated after stimulation of naive CD8 T lymphocytes. Again, when naive CD8 T lymphocytes were activated in the presence of hAMSCs, their gene expression profile, and in particular *SREBP1* and *2*, *PDK1*, and *RAPTOR* genes, was comparable to that of unstimulated naive CD8 T lymphocytes. Similarly, the expression of genes involved in the epigenetic response such as *BACH2* and *ID3* or the transcription factor *BCL6*^46^^,^^47^ was downregulated after activation and preserved in the presence of hAMSCs (Figures 5B and S3).

Finally, citrate synthase *(CS)*, hexokinase 2 (*HK2*), and *AKT* resulted low expressed in both unstimulated naive and activated CD8 T lymphocytes, while upregulated upon co-culture with hAMSCs (Figures 5C; Figure S3).

The results obtained emphasize that hAMSCs block CD8 cells that keeps them transcriptionally similar to naive CD8 by inhibiting some of the signaling pathways responsible for the activation and commitment of CD8 lymphocytes.

### hAMSCs regulate the downstream signaling cascade of IL-12 and IL-2 receptor

**Figure 6:**
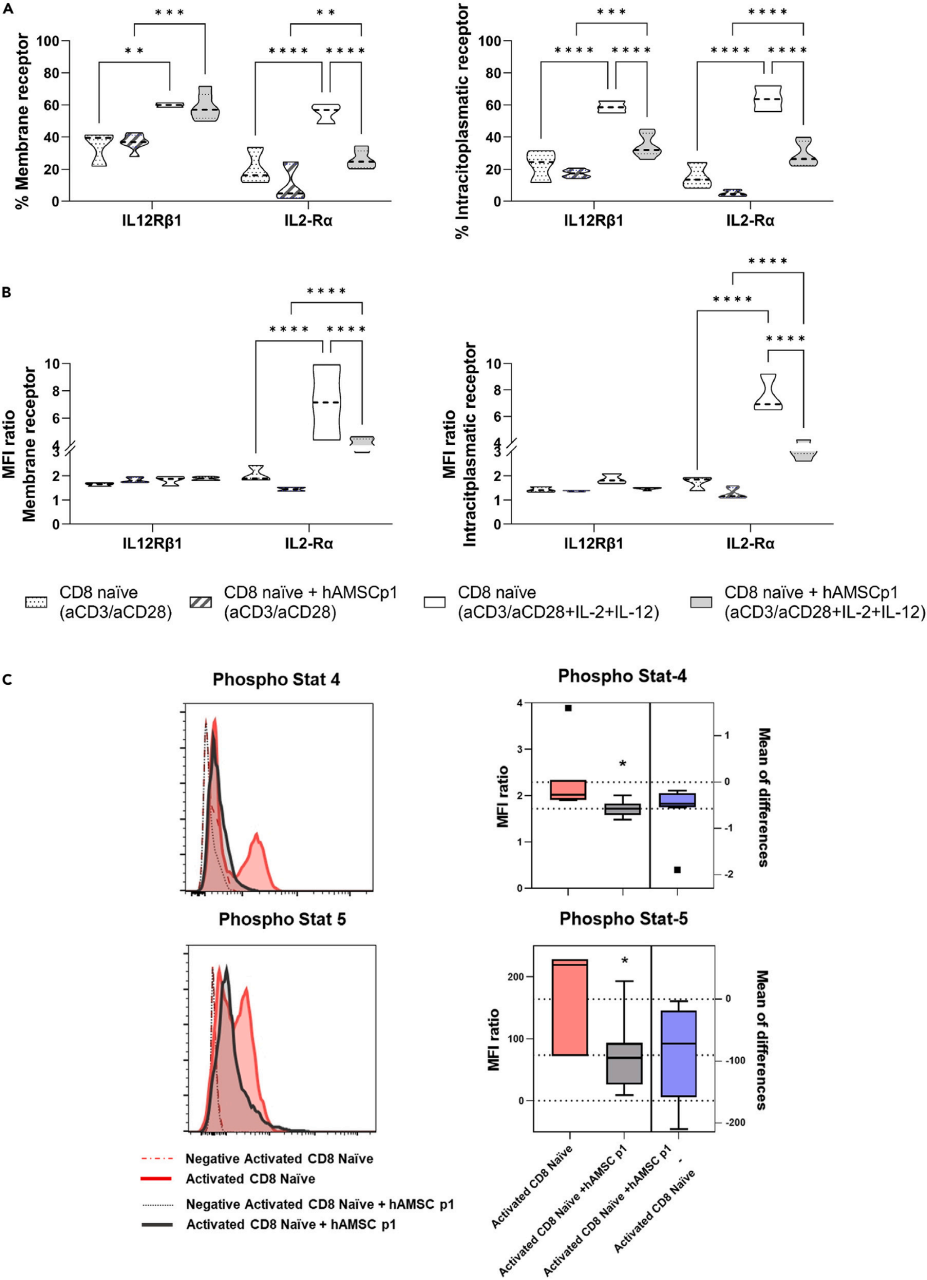
hAMSCs regulate the downstream signaling cascade of IL-12 and IL-2 receptor Purified naive CD8 T lymphocytes were either stimulated with anti CD3, anti CD28, and by the exogeneous administration of IL-12 and IL-2 or without the exogenous administration of the two cytokines (not fully activated) in the presence or not of hAMSCs. The total amount of IL-12Rβ1 and IL-2Rα expressed on the membrane and in the cytoplasm of CD8 T cells (A) and the level of mean fluorescence intensity (B) were assessed by flow cytometry. The phosphorylation status of the STAT4 and STAT5 was assessed by flow cytometry on fully activated naive CD8 T lymphocytes co-cultured or not with hAMSCs (C). Results are displayed as violin plots showing median (dashed line), 25th and 75th quartiles (∗p < 0.05, ∗∗p < 0.01∗∗∗∗p < 0.001); N = 4 independent experiments performed starting from 4 purified CD8 naive donors and 6 different hAMSC preparation.

Given the transcriptional downregulation of IL12Rβ1 on CD8 T cells after hAMSCs challenge (Figure 5A), we speculated on the possible involvement of this receptor in the mechanism of action of hAMSCs. To confirm our hypothesis, we examined the expression of the IL-2Rα and that of IL-12Rβ1 at day 3 on both the surface and cytoplasm of activated naive CD8 T lymphocytes after complete and incomplete stimulation (i.e., in the absence of exogenous IL-12 and IL-2 stimulation). As shown in Figure 6A, the expression of IL-12Rβ1 on the surface of activated naive CD8 lymphocytes increased after exogenous stimulation with the two cytokines, but no significant difference in IL-12Rβ1 was observed when CD8 T lymphocytes were co-cultured with hAMSCs when compared with naive CD8 T lymphocytes. These results were also confirmed by the MFI data (Figure 6B). Instead, a strong decrease in IL-2Rα expression was observed in CD8 lymphocytes co-cultured with hAMSCs (Figures 6A and 6B). Whereas this has been observed previously for IL2-Rα in CD4,^48^ this result is completely unknown for both IL2-Rα and IL-12Rβ1 on CD8 T lymphocytes. Indeed, the expression levels of IL-12Rβ1 in the membrane of naive CD8 cells co-cultured with hAMSCs (either incompletely or fully activated) were comparable to those of the control condition (naive CD8 T lymphocytes activated with the different combination of stimuli) (Figures 6A and 6B). This trend was not confirmed at the cytoplasmic level, where co-culture with hAMSCs resulted in a decrease in IL-12Rβ1 expression. These results are functionally reflected in a reduction in downstream signaling of the two receptors. Indeed, activation of naive CD8 T cells in the presence of hAMSCs reduced phosphorylation of STAT4 (downstream the IL-12Rβ1 receptor) and STAT5 (downstream the IL2-Rα receptor).

## Discussion

This study sheds light on the mechanism of action of MSCs isolated from the amniotic membrane (hAMSCs), by explaining how they affect CD8 T lymphocyte activation and differentiation and their commitment toward memory subsets. Indeed, it is well-established that MSCs possess the ability to inhibit the activation of T lymphocytes. Considerable knowledge exists regarding their capacity to direct the polarization of CD4 lymphocytes toward immunoregulatory subsets (Tregs), concomitantly leading to a reduction in the polarization of inflammatory subsets. However, not much is known about the capability of MSCs to influence the activation and differentiation of CD8 lymphocytes. Notably, in this study conducted on PBMCs, we observed that hAMSCs can attenuate the proliferation and the differentiation of stimulated T lymphocytes, promoting the preservation of T cells in a naive state. This effect may be partly attributed to the ability of hAMSCs to induce cell-cycle arrest, as previously reported by our group in the context of tumor cells.^49^ This cell-cycle arrest exerted by hAMSCs also influences the commitment of CD8 lymphocytes. Notably, hAMSCs induce a cessation in the expression of transcription factors T-bet and Eomes, both of which play critical roles in the differentiation process of T cells.^36^^,^^50^^,^^51^ Specifically, our study shows that hAMSCs block CD8 T cell differentiation and that this is related on the ability of hAMSCs to modulate the expression and downstream signaling of IL-12 and IL-2, receptors which are essential for the full activation and subsequent maturation of naive CD8 lymphocytes.^39^

Remarkably, we observed that hAMSCs do not counteract the activation of CD8 T lymphocytes, because after stimulation naive CD8 T lymphocytes are equally capable of expressing CD183, a marker that is rapidly induced on naive T lymphocytes after activation,^52^ but rather decrease commitment to the various memory subsets (CM, EM). This effect has been reported previously for both hAMSCs^25^ and bone marrow-derived MSC, although in the latter it was observed only after stimulation with the peptide HY.^53^

It is well known that cell activation and metabolic activity are closely related. Therefore, we investigated whether hAMSCs are able to modulate the PI3K pathway and consequently the activation of mTOR. The PI3K pathway is involved not only in the activation of T lymphocytes,^41^ but also in the regulation of metabolism,^54^ and mTOR is considered a master regulator of memory CD8^+^ T cell differentiation.^55^^,^^56^ We observed that hAMSCs are able to decrease the phosphorylation levels of AKT and mTOR, suggesting a possible mechanism to modulate the activation of CD8 T lymphocytes. However, this result is in contradiction with the results of transcriptional analysis, where we observed increased expression of AKT, mTOR, LDHA, CS, and HK2 all genes whose expression is directly involved in the regulation of the various metabolic pathways underlying T cell activation and early commitment.^57^ In the context of T cell receptor (TCR) stimulation, signals stemming from growth factor cytokines such as IL-2 and the binding of co-stimulatory CD28 receptors can lead to an upsurge in glycolysis. This increase is a result of the PI3K-dependent activation of Akt.^58^^,^^59^ This, in turn, can promote the activation of the mTOR pathway.^60^ Additionally, it can stimulate glycolysis by elevating the activity of glycolytic enzymes and enhancing the expression of nutrient transporters. This enables an enhanced utilization of glucose and amino acids needed to support the high rate of proliferation of activated cells.^61^^,^^62^^,^^63^ The involvement of fatty acid metabolism has also been known in the activation process. Sterol regulation during T cell activation has been linked to transcriptional responses mediated by SREBP and LXR.^64^

In our case, we observed levels of SREBP1 and 2 expressions comparable to those in naive CD8 lymphocytes. Interestingly, blocking the mTOR pathway gives effects similar to those we observed with hAMSCs. mTOR blockade, indeed inhibit cell proliferation while favoring the promotion of other forms of nutrient (protein) catabolism.^65^

In this regard, our findings suggest that hAMSCs may potentially impact CD8 naive T cell proliferation and activation by impeding cell cycle progression and even suggested by our Ki67 data, and by the reduced levels of total AKT and mTOR protein expression.

This effect may also be attributed to a metabolic reprogramming on multiple fronts. Supporting this hypothesis, for instance, is the expression level of AMPK, a protein involved in the regulation of lipid metabolism. In cells co-cultured with hAMSCs, AMPK exhibits a gene expression pattern akin to that of naive T cells. The sustained presence of AMPK could elucidate the decrease in mTOR phosphorylation. In fact, AMPK functions by phosphorylating TSC2, thereby impeding mTOR activation.^66^

Altogether, these findings suggest that hAMSCs do not completely block activation triggered by TCR and costimulatory molecules, but rather interfere with subsequent differentiation processes. By analyzing the expression of the two receptors involved in CD8 memory commitment, we observed that hAMSCs downregulate the expression of *IL12RB1* transcript while upregulating the expression of *IL2RA*.

Analysis of membrane receptors, however, gave us unexpected results. While we expected a decrease in phosphorylation of STAT5 as a consequence of translational blockade of IL2RA, the similarity of the levels of IL-12Rβ1 in naive CD8 T cells with or without hAMSCs conditioning suggests that there is no difference in STAT4 phosphorylation. Interestingly, we observed that STAT4 phosphorylation was reduced when naive CD8 T lymphocytes were co-cultured with hAMSCs.

This finding leads us to hypothesize that hAMSCs either induce a phenotype of CD8 T cells that is less responsive to exogenous stimulation or a potential blockade of epigenetic or cross-mediated activation pathways. Another possible explanation is related to the potential alteration in the expression of IL-12Rβ2 whose heterodimerization with IL-12Rβ1 is required for full activation of the receptor and subsequent phosphorylation of STAT4.^67^ Another possible target of hAMSCs activity could be the IL-23 receptor, which also heterodimerizes with IL-12Rβ1, leading to dual phosphorylation of STAT4 and STAT3 downstream of IL-12Rβ1 and IL-23, respectively.^67^^,^^68^^,^^69^ Moreover, hAMSCs may result in a non-fully functional IL -12Rβ1 activation, as previously reported,^69^ which can explain the observed reduction in signal transduction consequently impacting on the phosphorylation of STAT4.

Furthermore, the lack of IL-2Rα translation means that ligands, even when administered exogenously, do not trigger the transduction cascade, which in turn could explain the lower activation induced by hAMSCs.

The low activation status is also reflected in the lack of *IFNγ* and *TNFα* expression. Indeed, the *IFNγ* promoter is highly methylated in naive CD8 lymphocytes.^70^ hAMSCs likely retain this property in naive CD8 T lymphocytes, suggesting epigenetic regulation by hAMSCs that correlates with their immunomodulatory capacity.

Differentiation of naive CD8 lymphocytes is regulated by a dynamic and tightly coordinated process involving Tbet and Eomes^1^^,^^38^^,^^39^ as well as other transcription factors such as *IRF4*, *BCL6*, and epigenetic factors such as *BACH2*, and *ID3.*^37^^,^^71^^,^^72^ We observed that hAMSCs modulate this innate cellular commitment and, in particular, affect T lymphocyte activation and differentiation into SLEC and MPEC subsets. *TBX21* and *EOMES*, the two major regulators of SLEC/MPEC fate, are transcriptionally downregulated by hAMSCs as in nonactivated naive CD8 T cells. Consistently, protein expression of Tbet and Eomes was also decreased, which may be related to the observed reduction in STAT4 and STAT5 phosphorylation.^72^^,^^73^

Furthermore, hAMSCs are able to affect the expression of *BACH2* by decreasing the availability of the AP-1 complex at transcription sites thus inducing the repression of genes related to terminal differentiation of T lymphocytes.^74^

In addition, we observed that hAMSCs modulate the expression of *ID2* and *ID3*, two genes that have been reported to be involved in the regulation of SLEC or MPEC commitment.^46^ IL-2, IL-12, and IL-21 have been described to upregulate *ID2* and downregulate *ID3* expression. We show that hAMSCs impact the expression of *ID2* and *ID3* by reducing IL-2Rα and IL-12Rβ1 signal transduction, thus explaining the high level of *ID3* expression comparable to that of naive CD8 T lymphocytes, and at the same time the reduction of ID2 expression. Conversely, *ZEB2*, a target of Tbet normally involved in differentiation processes and induction of effector CD8 T lymphocyte differentiation,^75^ was upregulated by co-culture with hAMSCs.

Our work unravels an unknown immunological mechanism of hAMSCs that controls the transcriptional profile of CD8 T lymphocytes between that of naive and activated cells, thereby influencing the formation of an immunological memory. One of the major implications of our study is that it focuses on a poorly studied aspect that is mostly neglected in the field of MSC, namely the effects of MSC on CD8 lymphocytes. Although MSC remain one of the most studied cell therapies for the treatment of immune-related diseases due to their immunoregulatory capabilities, it remains critical to understand what will occur in a patient’s immune system and if the treatment may have side effects. In addition, we report a mechanism of action for hAMSCs to explain the observed effect.

Moreover, it supports the possibility of administering multiple doses of MSC, even from different donors, and highlights their therapeutic potential in graft-versus-host disease^6^ or autoimmune diseases in which CD8 T lymphocytes play an important role.^76^^,^^77^

### Limitations of the study

Our study, provides insights into the immunomodulatory capabilities of amniotic MSCs, particularly in modulating the activation and commitment of naive CD8 lymphocytes toward memory subsets. Although our study has unveiled alternative mechanisms, there are some limitations to consider.

Firstly, the study was conducted through PBMC/purified CD8 naive T lymphocytes and hAMSCs in co-culture in direct contact. While we have previously observed immunomodulatory effects by utilizing both hAMSCs in transwell system and the hAMSC secretome, suggesting the involvement of secreted factors, we cannot exclude the possibility that some of the observed effects may be the consequence of specific interactions with surface receptors expressed by hAMSCs.

Secondly, our study does not definitively identify the specific factor/s responsible for this immunomodulatory action. This is indeed a challenging task given the multitude of factors and molecules that could potentially contribute to such an action. Finally, another potential limitation could be that the study was conducted with a specific MSC population from the amniotic membrane, raising the possibility that the mechanism described here may be specific to these cells and not applicable to MSC from different tissues. Conducting a comparative study would be valuable in elucidating whether this mechanism is a shared characteristic among all types of MSCs. This clarification holds significance, especially in light of their widespread utilization in clinical trials, and it could provide guidance for their application in the treatment of conditions with distinct characteristics. However, this research is crucial for advancing our comprehension of the therapeutic potential of these cells and for more precisely delineating their capacities in translational applications.

## STAR★Methods

### Key resources table

**Table:** 

REAGENT or RESOURCE	SOURCE	IDENTIFIER (clone)
**Antibodies**		

CD3 BV480	BD Biosciences	UCHT1, Cat# 566105; RRID:AB_2739507
CD8 BV421	BD Biosciences	RPA-T8, Cat# 562428; RRID:AB_11154035
CD197/CCR7 Alexa Fluor® 647	BD Biosciences	3D12, Cat# 557734; RRID:AB_396842
CD45RO PE-CF594	BD Biosciences	UCHL1, Cat# 562299; RRID:AB_11154398
CD27 PerCP-Cy5.5	BD Biosciences	M-T271, Cat# 560612; RRID:AB_1727457
CD183/CXCR3 PE	BD Biosciences	1C6/CXCR3,Cat# 557185; RRID:AB_396596
KLRG1 PE-Vio770	Miltenyi Biotec	REA261, Cat# 130-120-428; RRID:AB_2784409
CD212/IL12Rβ1 BV605	BD Biosciences	2.4E6, Cat# 744204; RRID:AB_2742056
CD25/IL2RA BUV563	BD Biosciences	2A3, Cat# 612918; RRID:AB_2870203
Ki67+ Alexa 488	BD Biosciences	B56, Cat# 561165; RRID:AB_10611866
Tbet APC	Miltenyi Biotec	REA102, Cat# 130-098-607; RRID:AB_2653649
Eomes FITC	Invitrogen	WD1928, Cat# 11-4877-42 (also 11-4877); RRID:AB_2572499
AKT	Cell Signaling	C6E7, Cat# 9272 (also 9272S); RRID:AB_329827
mTOR	Cell Signaling	Catalog number, Cat# 2972 (also 2972S); RRID:AB_330978
Secondary antibody fluorochrome- conjugated anti-Rabbit Dylight 488	Vector Laboratories	Catalog number DI-1088; RRID:AB_2336403
Stat4 (pY693) Alexa fluor® 488	BD Biosciences	38/p-Stat4, Cat# 558136; RRID:AB_397051
Stat5 (pY694) PE-Cy7	BD Biosciences	47/Stat5(pY694), Cat# 560117; RRID:AB_1645546
Akt (pS473) PE-CF594	BD Biosciences	M89-61, Cat# 562465; RRID:AB_2737620
mTOR (pS2448) Alexa fluor® 647	BD Biosciences	021-404, Cat# 564242; RRID:AB_2738695

**Biological samples**		

Human peripheral blood mononuclear cells from healthy donor		
Amniotic mesenchymal stromal cells from healthy donor		

**Chemicals, peptides, and recombinant proteins**		

Chang medium C	Irvine	T101-019
FBS	Merck	F9665
DMSO	Merck	D2650
UltraCULTURE™	Lonza	12-725F
Penicillin-Streptomycin	Sigma Aldrich	P0781
L-glutamine	Sigma Aldrich	G7513
RPMI 1640	Euroclone	ECB2000
Penicillin-Streptomycin	Euroclone	ECB3001
L-glutamine	Euroclone	ECB3000
IL-2	Miltenyi Biotec	130-097-743
IL-12	Miltenyi Biotec	130-096-705
Cytofix/Cytoperm	BD Biosciences	554714
Methanol-free formaldehyde	Thermo Fisher Scientific	28908
PBS	Sigma Aldrich	D5652
Transcription Factor buffer kit	BD Biosciences	562574
Histopaque	Sigma Aldrich	10771

**Deposited data**		

All data reported in this paper will be shared by the lead contact upon request.		
Any additional information required to reanalyze the data reported in this paper is available from the lead contact upon request.		

**Oligonucleotides**		

*AKT*	5′-TCTATGGCGCTGAGATTGTG-3′	5′-CTTAATGTGCCCGTCCTTGT-3′
*HK2*	5′-ATTGTCCAGTGCATCGCGGA-3′	5′-AGGTCAAACTCCTCTCGCCG-3′
*MTOR*	5′-CTGCTTCCTCGGACAACC-3′	5′-GACAACAGCCTTCTGGTGGC-3′
*RPS6KB1*	5′-TACTTCGGGTACTTGGTAA-3′	5′-GATGAAGGGATGCTTTACT-3′
*RAPTOR*	5′-ACTGATGGAGTCCGAAATG-3′	5′-TCATCCGATCCTTCATCCTC-3′
*RICTOR*	5′-GGAAGCCTGTTGATGGTGA-3′	5′-GGCAGCCTGTTTTATGGTGT-3′
*SREBF1*	5′-GCCATGGATTGCACTTT-3′	5′-CAAGAGAGGAGCTCAATG-3′
*SREBF2*	5′-AGGCAGGCTTTGAAGACGAA-3′	5′-GTACATCGGAACAGGCGGAT-3′
*PRKAA*	5′-TTGAAACCTGAAAATGTCCTGCT-3′	5′-GGTGAGCCACAACTTGTTCTT-3′
*CITRATE SYNTHASE*	5′-GATTGTGCCCAATGTCCTCT-3′	5′-TTCATCTCCGTCATGCCATA-3′
*LDHA*	5′-AACATGGCAGCCTTTTCCTT-3′	5′-TAAGACGGCTTTCTCCCTCT-3′
*MYC*	5′-TGAGGAGACACCGCCCAC-3′	5′-CAACATCGATTTCTTCCTCATCTTC-3′
*PDK1*	5′-CTGGGTAATGAGGATTTGACTGT-3′	5′-AAGTCTGTCAATTTTCCTCAAAGG-3′
*EOMES*	5′-AGGCGCAAATAACAACAACACC-3′	5′-ATTCAAGTCCTCCACGCCATC-3′
*TBX21*	5′-ACTGCCCCCAAGGAATTGAC-3′	5′-CCGTGACTGCCTACCAGAAT-3′
*FOXO1*	5′-TTATGACCGAACAGGATGATCTTG-3′	5′-TGTTGGTGATGAGAGAAGGTTGAG-3′
*IRF4*	5′-ACCGAAGCTGGAGGGACTAC-3′	5′-GTGGGGCACAAGCATAAAAG-3′
*TCF1*	5′-CTGACCTCTCTGGCTTCTACTC-3′	5′-CAGAACCTAGCATCAAGGATGGG-3′
*IL2RA*	5′-AATGCAGCCAGTGGACCAA-3′	5′-TGATAAATTCTCTCTGTGGCTTCATTT-3′
*STAT3*	5′-GGCCCCTCGTCATCAAGA-3′	5′-TTTGACCAGCAACCTGACTTTAGT-3′
*STAT4*	5′-CAGTGAAAGCCATCTCGGAGGA-3′	5′-TGTAGTCTCGCAGGATGTCAGC-3′
*STAT5*	5′-GCCACTGTTCTCTGGGACAATG-3′	5′-ACACGAGGTTCTCCTTGGTCAG-3′
*BACH2*	5′-CTGCCGCAAAAGGAAACTGGAC-3′	5′-GGAAAGGCAGGAGAAGTTGTCC-3′
*BCL6*	5′-CATGCAGAGATGTGCCTCCACA-3′	5′-TCAGAGAAGCGGCAGTCACACT-3′
*ID2*	5′-TTGTCAGCCTGCATCACCAGAG-3′	5′-AGCCACACAGTGCTTTGCTGTC-3′
*ID3*	5′-CAGCTTAGCCAGGTGGAAATCC-3′	5′-GTCGTTGGAGATGACAAGTTCCG-3′
*PRF1*	5′-ACTCACAGGCAGCCAACTTTGC-3′	5′-CTCTTGAAGTCAGGGTGCAGCG-3′
*ZEB1*	5′-GGCATACACCTACTCAACTACGG-3′	5′-TGGGCGGTGTAGAATCAGAGTC-3′
*ZEB2*	5′-AATGCACAGAGTGTGGCAAGGC-3′	5′-TGCTGATGTGCGAACTGTAGG-3′
*IL12Rβ1*	5′-GGAGTGAATCATTGAGAGCACAA-3′	5′-TGCCGTTTCATGTACCAGAC-3′
*IFNγ*	5′-GAGTGTGGAGACCATCAAGGAAG-3′	5′-TGCTTTGCGTTGGACATTCAAGTC-3′
*TNFα*	5′-CTCTTCTGCCTGCTGCACTTTG-3′	5′-ATGGGCTACAGGCTTGTCACTC-3′
*β2M*	5′-TTCTGGCCTGGAGGCTATC-3′	5′-TCAGGAAATTTGACTTTCCATTC-3′
*β-ACTIN*	5′-GGATGCAGAAGGAGATCACTG-3′	5′-CGATCCACACGGAGTACTTG-3′

**Software and algorithms**		

Prism 9	GraphPad	https://www.graphpad.com/features
FlowJo 10.8	FlowJo BD	https://www.flowjo.com/
Biorad CFX Maestro 2.2	Biorad	https://www.bio-rad.com/it-it/sku/12013758-cfx-maestro-software-2-3-for-windows-pc?ID=12013758
BD FACSDiva™ Software	BD	https://www.bdbiosciences.com/en-it/products/software/instrument-software/bd-facsdiva-software

**Other**		

SYBR green master mix	Biorad	1725272
iScript Advanced cDNA Synthesis Kit	Biorad	1725038
SsoAdvanced PreAmp Supermix	Biorad	1725160
Dynabeads human T-activator CD3/CD28	Thermo Fisher Scientific	11161D
eBioscienceTM Fixable Viability Dye eFluorTM 780	Thermo Fisher Scientific	65-0865-18
BD Trucount™ Trucount Absolute Counting Tubes IV	BD Biosciences	340334
Naïve CD8^+^ T Cell Isolation Kit	Miltenyi Biotec	130-093-244
MACS® separation columns	Miltenyi Biotec	130-042-201

### Resource availability

#### Lead contact

Further information and requests for resources and reagents should be directed to and will be fulfilled by the lead contact, Andrea Papait (andrea.papait@unicatt.it).

#### Materials availability

This study did not generate new unique reagents.

#### Data and code availability

• All data reported in this paper will be shared by the lead contact upon request.
• This paper does not report original code.
• Any additional information required to reanalyze the data reported in this paper is available from the lead contact upon request

### Experimental model and study participant details

#### Human specimens

The collection of human peripheral blood mononuclear cells (PBMC) for research purposes was approved by the Regional Departments of Transfusion Medicine and after obtaining informed written consent, according to the guidelines set by the local ethical committee “Comitato Etico Provinciale di Brescia,” Italy (NP 3968, July 2, 2020). PBMC were obtained from healthy adult donors.

Human term placentae were collected from healthy women after vaginal delivery or caesarean section at term, after obtaining informed written consent, according to the guidelines set by the local ethical committee “Comitato Etico Provinciale di Brescia,” Italy (number NP 2243, January 19, 2016).

#### Isolation and culture of human amniotic mesenchymal Stromal Cells

Placentas were processed immediately after collection and human amniotic mesenchymal stromal cells (hAMSCs) isolated as previously described^77^ Then the cells were plated and expanded until passage 1 (hAMSCs p1) at a density of 1 x10^4^/cm2 in Chang medium C (Irvine Scientific, Santa Ana, CA, USA) supplemented with 2 mM L-glutamine at 37°C in the incubator at 5% CO2. Upon reaching confluence, adherent cells were trypsinized and frozen in fetal bovine serum (FBS, Merck, St. Louis, MO, USA) with 10% DMSO (Merck) and stored in liquid nitrogen. hAMSCs p1 were phenotypically characterized as previously reported.^77^ Cells that had >98% expression of mesenchymal markers CD13 and CD90, <2% of hematopoietic marker CD45 and <2% of epithelial marker CD324 were used in this study.

#### Isolation of PBMC and naive CD8 T lymphocytes

PBMC were separated from Buffy Coat through density gradient centrifugation (Histopaque, Sigma- Aldrich,St. Louis, MO, USA), then frozen in FBS with 10% DMSO (Merck) and stored in liquid nitrogen. Naive CD8 T lymphocytes were purified from total PBMC by Naive CD8^+^ T Cell Isolation Kit and MACS® separation columns (Miltenyi Biotec, Bergisch Gladbach, Germany), following manufacturer’s instructions. Naive CD8^+^ T lymphocyte was analyzed with flow cytometry by CD3, CD8, CD197/CCR7, CD45RO expression and the purity resulted >95-98% of the total cells recovered. For the molecular analysis 1 × 10^6^ Naive CD8 lymphocytes were centrifuged and pellets were stored at −80°C before the RNA extraction.

### Method details

#### Activation of T cells in PBMC and co-culture with hAMSCs

PBMC (1 × 10^5^/well in a 96-well-plate) were seeded in UltraCULTURE™ complete medium, composed of UltraCULTURE™ medium (Lonza, Basel, Switzerland), supplemented with 2 mM L-glutamine and 1% P/S (both from Sigma-Aldrich) and they were activated with 125 ng/mL (final concentration) anti-CD3 (clone OKT3, BD Biosciences, Franklin Lakes, New Jersey, USA).

hAMSCs p1 were plated in RPMI complete medium (composed of RPMI 1640 medium supplemented with 10% heat-inactivated fetal bovine serum (FBS), 1% penicillin and streptomycin, and 1% L-glutamine (all from Euroclone, Pero, MI, Italy), left to adhere overnight (O/N) and γ-irradiated at 30Gy to block their proliferation. Activated PBMC (PBMC + anti-CD3) were cultured in the presence (in contact) or absence (control condition) of 1 × 10^5^ hAMSCs p1 in UltraCULTURE™ complete medium. Flow cytometry analysis was performed at day 3 and 7.

#### Activation and differentiation of naive CD8 T lymphocytes and co-culture with hAMSCs

Naive CD8 T lymphocytes (1 × 10^5^/well in a 96-well-plate, and 1 × 10^6^/well in a 24-well-plate) were seeded in UltraCULTURE™ complete medium and partially activated with dynabeads human T-activator CD3/CD28 (final dilution 1:1000) (Thermo Fisher Scientific, Waltham, MA USA) or completely activated with dynabeads human T-activator CD3/CD28 and IL-2 2.5 U/ml, IL-12 10 ng/ml (both from Miltenyi Biotec, Bergisch Gladbach, Germany).

hAMSCs p1 were plated in RPMI complete medium, left to adhere O/N and γ-irradiated at 30Gy. Naive CD8 T lymphocytes (complete or partially activated) were cultured in the presence (in contact) or absence (control condition) of hAMSCs p1 (1 × 10^5^/well in a 96-well-plate, and 1 × 10^6^/well in a 24-well- plate) in UltraCULTURE™ complete medium. Flow cytometry analysis was performed at day 3, 7 and 10.

#### Flow cytometry analysis of CD8 T lymphocyte polarization

Cells were collected at day 3 and 7 (PBMC) and at day 3, 7, and 10 (naive CD8 T lymphocytes), and cells were stained with the eBioscienceTM Fixable Viability Dye eFluorTM 780 (Thermo Fisher Scientific) according to the manufacturer’s instructions, in order to exclude dead cells during analysis.

Cells were subsequently stained for 20 min a 4°C with the appropriate combinations of fluorochrome-conjugated anti-human antibodies for the identification of CD8 T cells and sub-populations: CD3 BV480 (clone UCHT1, BD Biosciences), CD8 BV421 (clone RPA-T8, BD Biosciences), CD197/CCR7 Alexa Fluor® 647 (clone 3D12, BD Biosciences), CD45RO PE-CF594 (clone UCHL1, BD Biosciences), CD27 PerCP-Cy5.5 (clone M-T271, BD Biosciences), CD183/CXCR3 PE (clone 1C6/CXCR3, BD Biosciences), KLRG1 PE-Vio770 (clone REA261, Miltenyi), CD212/IL12Rβ1 BV605 (clone 2.4E6, BD Biosciences) and CD25/IL2RA BUV563 (clone 2A3, BD Biosciences).

After membrane staining, intracellular staining with CD212/IL12Rβ1 and CD25/IL2RA was performed after fixation and permeabilization using BD Cytofix/Cytoperm (BD Biosciences).

Proliferation of the T lymphocytes was assessed with Ki67+ Alexa 488 (clone B56, BD Biosciences) and Tbet APC (clone REA102, Miltenyi) and Eomes FITC (clone WD1928, Invitrogen) were assessed by staining with conjugated monoclonal antibody after intranuclear permeabilization used Transcription Factor buffer kit (BD). CD8 absolute count was performed by using BD Trucount™ Trucount Absolute Counting Tubes IV (BD Bioscences) according to manufacturer instruction.

Cells were collected at day 3 and 7 (PBMC) and at day 3, 7, and 10 (naive CD8 T lymphocytes), and cells were stained with the eBioscienceTM Fixable Viability Dye eFluorTM 780 (Thermo Fisher Scientific) according to the manufacturer’s instructions, in order to exclude dead cells during analysis.

For the analysis conducted on CD8 T lymphocytes to identify naive to memory subsets, the gating strategy was as follows: after initial gating based on morphological characteristics, we selected live cells as eFluorTM 780 dim/negative cells. Within the live cell population, we excluded doublets. Subsequently, we gated for CD3 and CD8-positive cells. The dissection of naive to memory subsets was performed based on the differential expression of CD45RO and CD197 markers, defining the subsets as follows: Naive cells: CD45RO-CD197+, Central Memory (CM): CD45RO+CD197+, Effector Memory (EM): CD45RO+CD197-, Terminally Differentiated Effector Memory (TEMRA): CD45RO-CD197-.

The gating strategy for distinguishing Short-Lived Effector Cells (SLEC) and Memory Precursor Effector Cells (MPEC) started with the gate on CD8 lymphocytes, as previously described. We further dissected these subsets based on the differential expression of CD183 and KLRG1 markers, defining the subsets as follows: Short-Lived Effector Cells (SLEC): CD183-KLRG1+, Early Effector Cells (EEC): CD183+KLRG1+, Memory Precursor Effector Cells (MPEC): CD183+KLRG1-, Double Negative Effector Cells (DNEC): CD183-KLRG1-.

Unsupervised analyses on naive CD8 lymphocytes was performed to investigate cell fate development at different time points (day 3, 7, and 10). Multidimensional analysis, was performed by using FlowSOM meta- clustering in conjunction with a dimensionality reduction method such as Uniform Manifold Approximation and Projection (UMAP). The identification of specific cluster was performed by using Cluster Explorer plugin.

Samples were acquired on FACS Symphony A3 BD. Data were analyzed with FlowJo 10.8.

#### Determination of phosphorylation status

Three days after co-culture the cells were fixed by adding 1.5% methanol-free formaldehyde (ThermoFisher) for 10 min at room temperature (RT). Then, the cells were collected and stained for 20 min at 4°C with CD8 BV421 (RPA-T8, BD Biosciences). After cell permeabilization in cold 90% methanol for 2–3 days at −80°C, cells were stained in the dark with total AKT and total mTOR (both of Cell signaling Technology, Danvers, Massachusetts, USA) for 30 min at RT, then the cells were washed in stain buffer, consisting of 0.02% sodium azide and 0.1% bovine serum albumin in PBS (Sigma-Aldrich). Cells were then incubated for 30 min at RT with secondary antibody fluorochrome- conjugated anti-Rabbit Dylight 488 (Vector Laboratories, Newark, CA, USA). Finally, cells were stained for 1h at RT with BD PhosflowTM (BD Bioscience) fluorescently conjugated antibodies against: Stat4 (pY693) Alexa fluor® 488 (clone 38/p-Stat4), Stat5 (pY694) PE-Cy7 (clone 47/Stat5(pY694)), Anti-Akt (pS473) PE-CF594 (clone M89-61), Anti-mTOR (pS2448) Alexa fluor® 647 (clone 021-404) all purchased from BD Biosciences.

Samples were acquired on FACS Symphony A3 BD. Data were analyzed with FlowJo 10.8.

#### Quantitative real-time PCR

Total RNA was extracted using EZ1 RNA cell Mini Kit protocol (Qiagen, Frederick, MD, USA), in a BioRobot EZ1 Advanced XL Workstation. The iScript Advanced cDNA Synthesis Kit for RTqPCR (Biorad, Hercules, California, USA) was used for cDNA synthesis, before quantitative real-time PCR cDNA was pre-amplified with SsoAdvanced PreAmp Supermix (Biorad). Real-time PCR was performed using the Biorad instrument CFX96 Quantitative Real-Time PCR. Data were analyzed with Biorad CFX Maestro 2.2 (Biorad). For molecular analysis, cells were collected at day 1 and CD8^+^ T cells were purified from hAMSCs by positive selection using CD8 microbeads and MACS® separation columns (Miltenyi) according to the manufacturer’s instructions. After separation, the cells were centrifuged and pellets were stored at −80°C for RNA extraction.

### Quantification and statistical analysis

The sample size calculation will be set based on previous literature (*53*) in which the effect of hAMSC secretome on immunomodulatory activity was evaluated. In detail, in such a study the use of hAMSC secretome in PBMC reduced the lymphocytes proliferation mean from 4.6 (x10^4^cpm) (SD about 1.4) to 1.5 (SD = 1.2). Consequently, the sample size of the PBMC study was of ≥3 healthy donors for each readout performed. Data are shown as violin truncated plots with Tukey variations. The parameters were compared using two-way, one-way analysis of variance (ANOVA) and Student’s *t* test. Normal distribution was analyzed by performing a Q-Q probability plot—and analytically by performing Shapiro–Wilk test, Kolmogorov–Smirnov test. Statistical details of every experiment can be found in the figure legends, median and quartile and value of n. Statistical analysis was performed using Prism 9 (GraphPad Software, La Jolla, CA, USA). A p value lower than 0.05 was considered statistically significant.

## References

[1] Obar J.J., Lefrançois L. (2010). Early events governing memory CD8+ T-cell differentiation. Int. Immunol..

[2] Joshi N.S., Cui W., Chandele A., Lee H.K., Urso D.R., Hagman J., Gapin L., Kaech S.M. (2007). Inflammation directs memory precursor and short-lived effector CD8(+) T cell fates via the graded expression of T-bet transcription factor. Immunity.

[3] Reina-Campos M., Scharping N.E., Goldrath A.W. (2021). CD8(+) T cell metabolism in infection and cancer. Nat. Rev. Immunol..

[4] Benichou G., Gonzalez B., Marino J., Ayasoufi K., Valujskikh A. (2017). Role of Memory T Cells in Allograft Rejection and Tolerance. Front. Immunol..

[5] Collier J.L., Weiss S.A., Pauken K.E., Sen D.R., Sharpe A.H. (2021). Not-so-opposite ends of the spectrum: CD8(+) T cell dysfunction across chronic infection, cancer and autoimmunity. Nat. Immunol..

[6] Zhang Y., Joe G., Hexner E., Zhu J., Emerson S.G. (2005). Host-reactive CD8+ memory stem cells in graft-versus-host disease. Nat. Med..

[7] Squillaro T., Peluso G., Galderisi U. (2016). Clinical Trials With Mesenchymal Stem Cells: An Update. Cell Transplant..

[8] Rodríguez-Fuentes D.E., Fernández-Garza L.E., Samia-Meza J.A., Barrera-Barrera S.A., Caplan A.I., Barrera-Saldaña H.A. (2021). Mesenchymal Stem Cells Current Clinical Applications: A Systematic Review. Arch. Med. Res..

[9] Ramasamy R., Tong C.K., Seow H.F., Vidyadaran S., Dazzi F. (2008). The immunosuppressive effects of human bone marrow-derived mesenchymal stem cells target T cell proliferation but not its effector function. Cell. Immunol..

[10] Glennie S., Soeiro I., Dyson P.J., Lam E.W.F., Dazzi F. (2005). Bone marrow mesenchymal stem cells induce division arrest anergy of activated T cells. Blood.

[11] Meisel R., Zibert A., Laryea M., Göbel U., Däubener W., Dilloo D. (2004). Human bone marrow stromal cells inhibit allogeneic T-cell responses by indoleamine 2,3-dioxygenase-mediated tryptophan degradation. Blood.

[12] Krampera M., Le Blanc K. (2021). Mesenchymal stromal cells: Putative microenvironmental modulators become cell therapy. Cell Stem Cell.

[13] Aggarwal S., Pittenger M.F. (2005). Human mesenchymal stem cells modulate allogeneic immune cell responses. Blood.

[14] Selmani Z., Naji A., Zidi I., Favier B., Gaiffe E., Obert L., Borg C., Saas P., Tiberghien P., Rouas-Freiss N. (2008). Human leukocyte antigen-G5 secretion by human mesenchymal stem cells is required to suppress T lymphocyte and natural killer function and to induce CD4+CD25highFOXP3+ regulatory T cells. Stem Cell..

[15] Choi H., Lee R.H., Bazhanov N., Oh J.Y., Prockop D.J. (2011). Anti-inflammatory protein TSG-6 secreted by activated MSCs attenuates zymosan-induced mouse peritonitis by decreasing TLR2/NF-κB signaling in resident macrophages. Blood.

[16] Galleu A., Riffo-Vasquez Y., Trento C., Lomas C., Dolcetti L., Cheung T.S., von Bonin M., Barbieri L., Halai K., Ward S. (2017). Apoptosis in mesenchymal stromal cells induces in vivo recipient-mediated immunomodulation. Sci. Transl. Med..

[17] Hass R., Kasper C., Böhm S., Jacobs R. (2011). Different populations and sources of human mesenchymal stem cells (MSC): A comparison of adult and neonatal tissue-derived MSC. Cell Commun. Signal..

[18] Zhang W., Ling Q., Wang B., Wang K., Pang J., Lu J., Bi Y., Zhu D. (2022). Comparison of therapeutic effects of mesenchymal stem cells from umbilical cord and bone marrow in the treatment of type 1 diabetes. Stem Cell Res. Ther..

[19] Rossi D., Pianta S., Magatti M., Sedlmayr P., Parolini O. (2012). Characterization of the conditioned medium from amniotic membrane cells: prostaglandins as key effectors of its immunomodulatory activity. PLoS One.

[20] Silini A.R., Magatti M., Cargnoni A., Parolini O. (2017). Is Immune Modulation the Mechanism Underlying the Beneficial Effects of Amniotic Cells and Their Derivatives in Regenerative Medicine?. Cell Transplant..

[21] Magatti M., De Munari S., Vertua E., Gibelli L., Wengler G.S., Parolini O. (2008). Human amnion mesenchyme harbors cells with allogeneic T-cell suppression and stimulation capabilities. Stem Cell..

[22] Papait A., Vertua E., Magatti M. (2020). Mesenchymal Stromal Cells from Fetal and Maternal Placenta Possess Key Similarities and Differences: Potential Implications for Their Applications in Regenerative Medicine. Cells.

[23] Magatti M., Masserdotti A., Bonassi Signoroni P., Vertua E., Stefani F.R., Silini A.R., Parolini O. (2020). B Lymphocytes as Targets of the Immunomodulatory Properties of Human Amniotic Mesenchymal Stromal Cells. Front. Immunol..

[24] Papait A., Ragni E., Cargnoni A., Vertua E., Romele P., Masserdotti A., Perucca Orfei C., Signoroni P.B., Magatti M., Silini A.R. (2022). Comparison of EV-free fraction, EVs, and total secretome of amniotic mesenchymal stromal cells for their immunomodulatory potential: a translational perspective. Front. Immunol..

[25] Pianta S., Bonassi Signoroni P., Muradore I., Rodrigues M.F., Rossi D., Silini A., Parolini O. (2015). Amniotic membrane mesenchymal cells-derived factors skew T cell polarization toward Treg and downregulate Th1 and Th17 cells subsets. Stem Cell Rev..

[26] Magatti M., De Munari S., Vertua E., Nassauto C., Albertini A., Wengler G.S., Parolini O. (2009). Amniotic mesenchymal tissue cells inhibit dendritic cell differentiation of peripheral blood and amnion resident monocytes. Cell Transplant..

[27] Magatti M., Vertua E., De Munari S., Caro M., Caruso M., Silini A., Delgado M., Parolini O. (2017). Human amnion favours tissue repair by inducing the M1-to-M2 switch and enhancing M2 macrophage features. J. Tissue Eng. Regen. Med..

[28] Cargnoni A., Piccinelli E.C., Ressel L., Rossi D., Magatti M., Toschi I., Cesari V., Albertini M., Mazzola S., Parolini O. (2014). Conditioned medium from amniotic membrane-derived cells prevents lung fibrosis and preserves blood gas exchanges in bleomycin-injured mice-specificity of the effects and insights into possible mechanisms. Cytotherapy.

[29] Cargnoni A., Romele P., Bonassi Signoroni P., Farigu S., Magatti M., Vertua E., Toschi I., Cesari V., Silini A.R., Stefani F.R., Parolini O. (2020). Amniotic MSCs reduce pulmonary fibrosis by hampering lung B-cell recruitment, retention, and maturation. Stem Cells Transl. Med..

[30] SantAnna L.B., Hage R., Cardoso M.A.G., Arisawa E.A.L., Cruz M.M., Parolini O., Cargnoni A., SantAnna N. (2016). Antifibrotic Effects of Human Amniotic Membrane Transplantation in Established Biliary Fibrosis Induced in Rats. Cell Transplant..

[31] Giampà C., Alvino A., Magatti M., Silini A.R., Cardinale A., Paldino E., Fusco F.R., Parolini O. (2019). Conditioned medium from amniotic cells protects striatal degeneration and ameliorates motor deficits in the R6/2 mouse model of Huntington's disease. J. Cell Mol. Med..

[32] Parolini O., Souza-Moreira L., O'Valle F., Magatti M., Hernandez-Cortes P., Gonzalez-Rey E., Delgado M. (2014). Therapeutic effect of human amniotic membrane-derived cells on experimental arthritis and other inflammatory disorders. Arthritis Rheumatol..

[33] Caplan A.I. (2017). Mesenchymal Stem Cells: Time to Change the Name. Stem Cells Transl. Med..

[34] Pittenger M.F., Discher D.E., Péault B.M., Phinney D.G., Hare J.M., Caplan A.I. (2019). Mesenchymal stem cell perspective: cell biology to clinical progress. NPJ Regen. Med..

[35] Gao F., Chiu S.M., Motan D.A.L., Zhang Z., Chen L., Ji H.L., Tse H.F., Fu Q.L., Lian Q. (2016). Mesenchymal stem cells and immunomodulation: current status and future prospects. Cell Death Dis..

[36] Hamilton S.E., Jameson S.C. (2007). CD8(+) T cell differentiation: choosing a path through T-bet. Immunity.

[37] Kaech S.M., Cui W. (2012). Transcriptional control of effector and memory CD8+ T cell differentiation. Nat. Rev. Immunol..

[38] Chowdhury F.Z., Ramos H.J., Davis L.S., Forman J., Farrar J.D. (2011). IL-12 selectively programs effector pathways that are stably expressed in human CD8+ effector memory T cells in vivo. Blood.

[39] Valenzuela J., Schmidt C., Mescher M. (2002). The roles of IL-12 in providing a third signal for clonal expansion of naive CD8 T cells. J. Immunol..

[40] Plumlee C.R., Obar J.J., Colpitts S.L., Jellison E.R., Haining W.N., Lefrancois L., Khanna K.M. (2015). Early Effector CD8 T Cells Display Plasticity in Populating the Short-Lived Effector and Memory-Precursor Pools Following Bacterial or Viral Infection. Sci. Rep..

[41] Kim E.H., Suresh M. (2013). Role of PI3K/Akt signaling in memory CD8 T cell differentiation. Front. Immunol..

[42] Pollizzi K.N., Patel C.H., Sun I.H., Oh M.H., Waickman A.T., Wen J., Delgoffe G.M., Powell J.D. (2015). mTORC1 and mTORC2 selectively regulate CD8⁺ T cell differentiation. J. Clin. Invest..

[43] Zhang L., Tschumi B.O., Lopez-Mejia I.C., Oberle S.G., Meyer M., Samson G., Rüegg M.A., Hall M.N., Fajas L., Zehn D. (2016). Mammalian Target of Rapamycin Complex 2 Controls CD8 T Cell Memory Differentiation in a Foxo1-Dependent Manner. Cell Rep..

[44] Salmond R.J. (2018). mTOR Regulation of Glycolytic Metabolism in T Cells. Front. Cell Dev. Biol..

[45] Nicoli F., Papagno L., Frere J.J., Cabral-Piccin M.P., Clave E., Gostick E., Toubert A., Price D.A., Caputo A., Appay V. (2018). Naïve CD8(+) T-Cells Engage a Versatile Metabolic Program Upon Activation in Humans and Differ Energetically From Memory CD8(+) T-Cells. Front. Immunol..

[46] Yang C.Y., Best J.A., Knell J., Yang E., Sheridan A.D., Jesionek A.K., Li H.S., Rivera R.R., Lind K.C., D'Cruz L.M. (2011). The transcriptional regulators Id2 and Id3 control the formation of distinct memory CD8+ T cell subsets. Nat. Immunol..

[47] Crotty S., Johnston R.J., Schoenberger S.P. (2010). Effectors and memories: Bcl-6 and Blimp-1 in T and B lymphocyte differentiation. Nat. Immunol..

[48] Yoo H.S., Lee K., Na K., Zhang Y.X., Lim H.J., Yi T., Song S.U., Jeon M.S. (2017). Mesenchymal stromal cells inhibit CD25 expression via the mTOR pathway to potentiate T-cell suppression. Cell Death Dis..

[49] Magatti M., De Munari S., Vertua E., Parolini O. (2012). Amniotic membrane-derived cells inhibit proliferation of cancer cell lines by inducing cell cycle arrest. J. Cell Mol. Med..

[50] Dejean A.S., Joulia E., Walzer T. (2019). The role of Eomes in human CD4 T cell differentiation: A question of context. Eur. J. Immunol..

[51] McLane L.M., Banerjee P.P., Cosma G.L., Makedonas G., Wherry E.J., Orange J.S., Betts M.R. (2013). Differential localization of T-bet and Eomes in CD8 T cell memory populations. J. Immunol..

[52] Groom J.R., Luster A.D. (2011). CXCR3 in T cell function. Exp. Cell Res..

[53] Krampera M., Glennie S., Dyson J., Scott D., Laylor R., Simpson E., Dazzi F. (2003). Bone marrow mesenchymal stem cells inhibit the response of naive and memory antigen-specific T cells to their cognate peptide. Blood.

[54] Saravia J., Raynor J.L., Chapman N.M., Lim S.A., Chi H. (2020). Signaling networks in immunometabolism. Cell Res..

[55] Araki K., Youngblood B., Ahmed R. (2010). The role of mTOR in memory CD8 T-cell differentiation. Immunol. Rev..

[56] Araki K., Turner A.P., Shaffer V.O., Gangappa S., Keller S.A., Bachmann M.F., Larsen C.P., Ahmed R. (2009). mTOR regulates memory CD8 T-cell differentiation. Nature.

[57] Braun M.Y. (2021). The Natural History of T Cell Metabolism. Int. J. Mol. Sci..

[58] Frauwirth K.A., Riley J.L., Harris M.H., Parry R.V., Rathmell J.C., Plas D.R., Elstrom R.L., June C.H., Thompson C.B. (2002). The CD28 signaling pathway regulates glucose metabolism. Immunity.

[59] Wieman H.L., Wofford J.A., Rathmell J.C. (2007). Cytokine stimulation promotes glucose uptake via phosphatidylinositol-3 kinase/Akt regulation of Glut1 activity and trafficking. Mol. Biol. Cell.

[60] Gingras A.C., Raught B., Sonenberg N. (2001). Regulation of translation initiation by FRAP/mTOR. Genes Dev..

[61] Barata J.T., Silva A., Brandao J.G., Nadler L.M., Cardoso A.A., Boussiotis V.A. (2004). Activation of PI3K is indispensable for interleukin 7-mediated viability, proliferation, glucose use, and growth of T cell acute lymphoblastic leukemia cells. J. Exp. Med..

[62] Elstrom R.L., Bauer D.E., Buzzai M., Karnauskas R., Harris M.H., Plas D.R., Zhuang H., Cinalli R.M., Alavi A., Rudin C.M., Thompson C.B. (2004). Akt stimulates aerobic glycolysis in cancer cells. Cancer Res..

[63] Rathmell J.C., Elstrom R.L., Cinalli R.M., Thompson C.B. (2003). Activated Akt promotes increased resting T cell size, CD28-independent T cell growth, and development of autoimmunity and lymphoma. Eur. J. Immunol..

[64] Bensinger S.J., Bradley M.N., Joseph S.B., Zelcer N., Janssen E.M., Hausner M.A., Shih R., Parks J.S., Edwards P.A., Jamieson B.D., Tontonoz P. (2008). LXR signaling couples sterol metabolism to proliferation in the acquired immune response. Cell.

[65] Peng T., Golub T.R., Sabatini D.M. (2002). The immunosuppressant rapamycin mimics a starvation-like signal distinct from amino acid and glucose deprivation. Mol. Cell Biol..

[66] Ma E.H., Poffenberger M.C., Wong A.H.T., Jones R.G. (2017). The role of AMPK in T cell metabolism and function. Curr. Opin. Immunol..

[67] Obar J.J., Jellison E.R., Sheridan B.S., Blair D.A., Pham Q.M., Zickovich J.M., Lefrançois L. (2011). Pathogen-induced inflammatory environment controls effector and memory CD8+ T cell differentiation. J. Immunol..

[68] Teng M.W.L., Bowman E.P., McElwee J.J., Smyth M.J., Casanova J.L., Cooper A.M., Cua D.J. (2015). IL-12 and IL-23 cytokines: from discovery to targeted therapies for immune-mediated inflammatory diseases. Nat. Med..

[69] Fieschi C., Bosticardo M., de Beaucoudrey L., Boisson-Dupuis S., Feinberg J., Santos O.F., Bustamante J., Levy J., Candotti F., Casanova J.L. (2004). A novel form of complete IL-12/IL-23 receptor beta1 deficiency with cell surface-expressed nonfunctional receptors. Blood.

[70] Lee P.P., Fitzpatrick D.R., Beard C., Jessup H.K., Lehar S., Makar K.W., Pérez-Melgosa M., Sweetser M.T., Schlissel M.S., Nguyen S. (2001). A critical role for Dnmt1 and DNA methylation in T cell development, function, and survival. Immunity.

[71] Chen Y., Zander R., Khatun A., Schauder D.M., Cui W. (2018). Transcriptional and Epigenetic Regulation of Effector and Memory CD8 T Cell Differentiation. Front. Immunol..

[72] Yang Y., Xu J., Niu Y., Bromberg J.S., Ding Y. (2008). T-bet and eomesodermin play critical roles in directing T cell differentiation to Th1 versus Th17. J. Immunol..

[73] Yu S.F., Zhang Y.N., Yang B.Y., Wu C.Y. (2014). Human memory, but not naive, CD4+ T cells expressing transcription factor T-bet might drive rapid cytokine production. J. Biol. Chem..

[74] Roychoudhuri R., Clever D., Li P., Wakabayashi Y., Quinn K.M., Klebanoff C.A., Ji Y., Sukumar M., Eil R.L., Yu Z. (2016). BACH2 regulates CD8(+) T cell differentiation by controlling access of AP-1 factors to enhancers. Nat. Immunol..

[75] Omilusik K.D., Best J.A., Yu B., Goossens S., Weidemann A., Nguyen J.V., Seuntjens E., Stryjewska A., Zweier C., Roychoudhuri R. (2015). Transcriptional repressor ZEB2 promotes terminal differentiation of CD8+ effector and memory T cell populations during infection. J. Exp. Med..

[76] Liblau R.S., Wong F.S., Mars L.T., Santamaria P. (2002). Autoreactive CD8 T cells in organ-specific autoimmunity: emerging targets for therapeutic intervention. Immunity.

[77] Silini A.R., Papait A., Cargnoni A., Vertua E., Romele P., Bonassi Signoroni P., Magatti M., De Munari S., Masserdotti A., Pasotti A. (2021). CM from intact hAM: an easily obtained product with relevant implications for translation in regenerative medicine. Stem Cell Res. Ther..

