# Supplementary material for: Amniotic MSC affect CD8 naive polarization toward SLEC/MPEC subsets by down-modulating IL-12Rβ1 and IL-2Rα signaling pathways
Source: iScience. 2023 Nov 17;26(12):108483. doi: 10.1016/j.isci.2023.108483 (PMC10709131; doi:10.1016/j.isci.2023.108483)
Supplement: Document S1. Figures S1–S3 [file mmc1.pdf]

iScience, Volume *26*

Supplemental information

Amniotic MSC affect CD8 naive polarization toward SLEC/MPEC subsets by down-modulating IL-12Rβ1 and IL-2Rα signaling pathways

Andrea Papait, Elsa Vertua, Patrizia Bonassi Signoroni, Anna Cargnoni, Marta Magatti, Francesca Romana Stefani, Jacopo Romoli, Antonietta Rosa Silini, and Ornella Parolini

Supplemental information

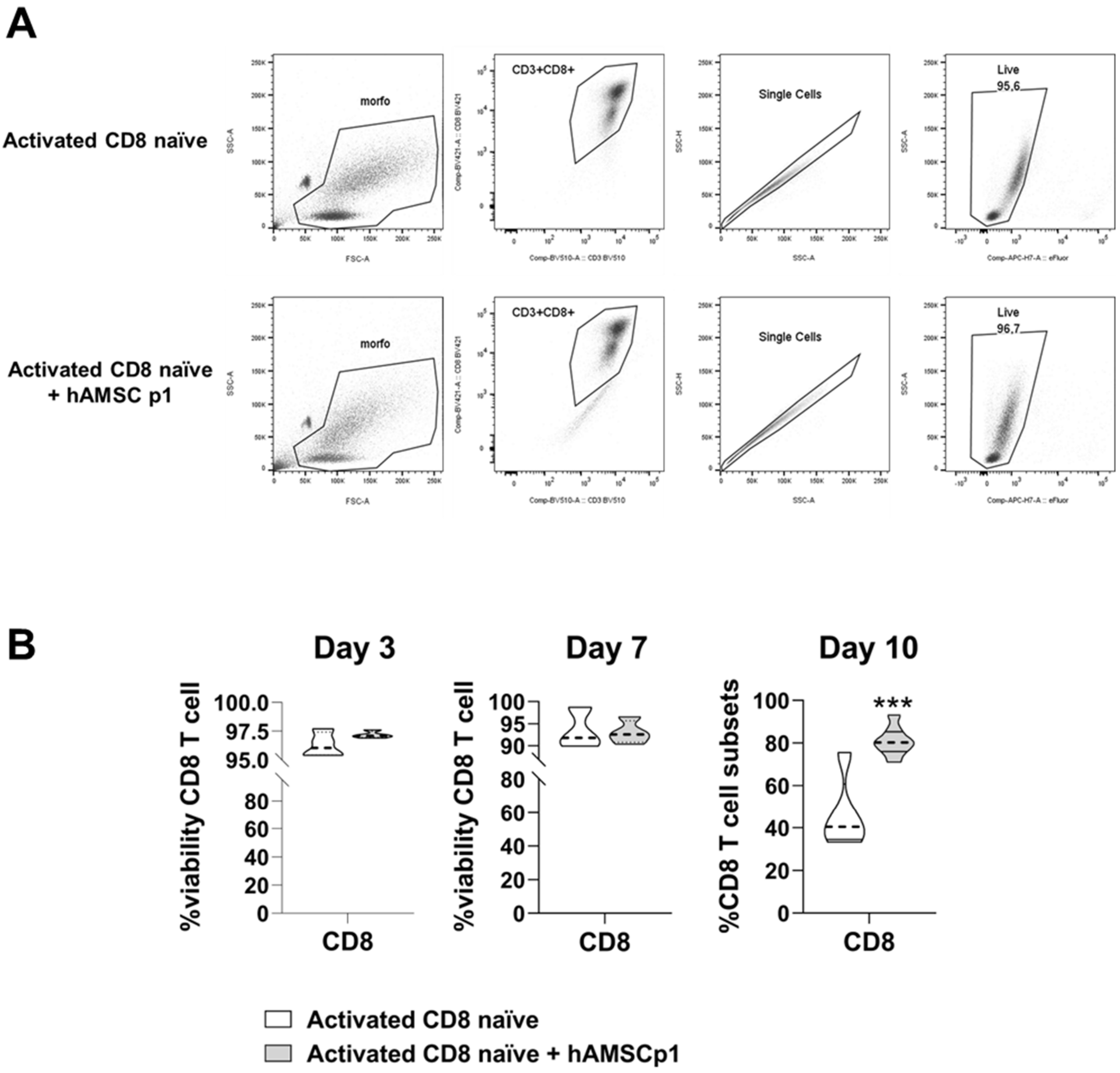

Figure S. 1. Cell viability

Representation of the gating strategy demonstrating how the analyses conducted were carried with live cells (A). Evaluation of viability at the three time points under study, 3, 7 and 10 days for activated CD8 naïve lymphocytes versus those activated and co-cultured in the presence of hAMSCs (A) Data are represented as dot plot. (B) Results are displayed as violin plots showing median (dashed line), 25th and 75th quartiles, ***$p < 0.001$.

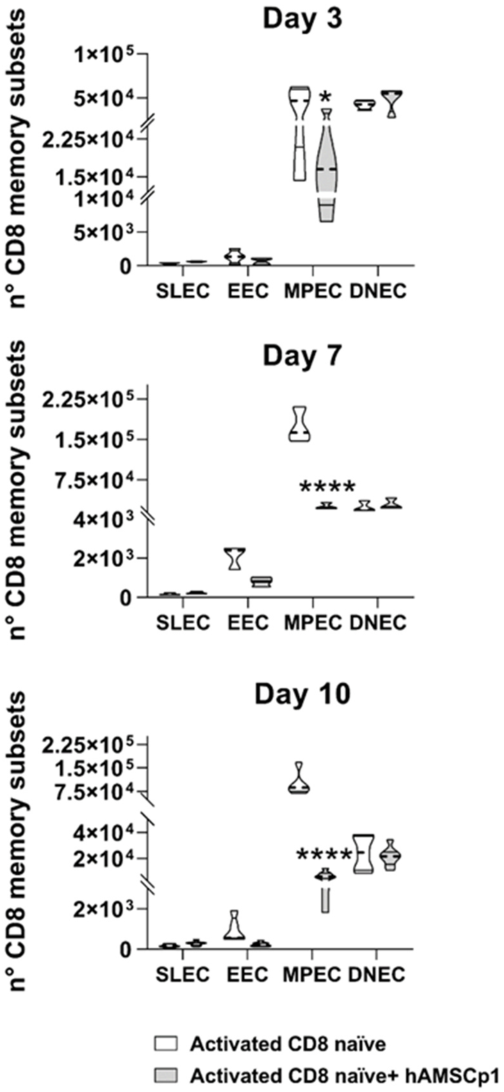

Figure S. 2. hAMSCs influence early phases of CD8 naïve T cell commitment
Purified CD8 naïve T lymphocytes were stimulated with antiCD3, antiCD28 and the exogeneous administration of IL12 and IL2, and cultured in the presence of hAMSCs (gray). CD8 T cells were allowed to differentiate for 10 days and the different cellular subtypes analyzed at day 3, 7 and 10 as absolute count for SLEC, MPED, EEC and DNEC subsets. Results are displayed as violin plots showing median (dashed line), 25th and 75th quartiles (*$p < 0.05$, ****$p < 0.001$)

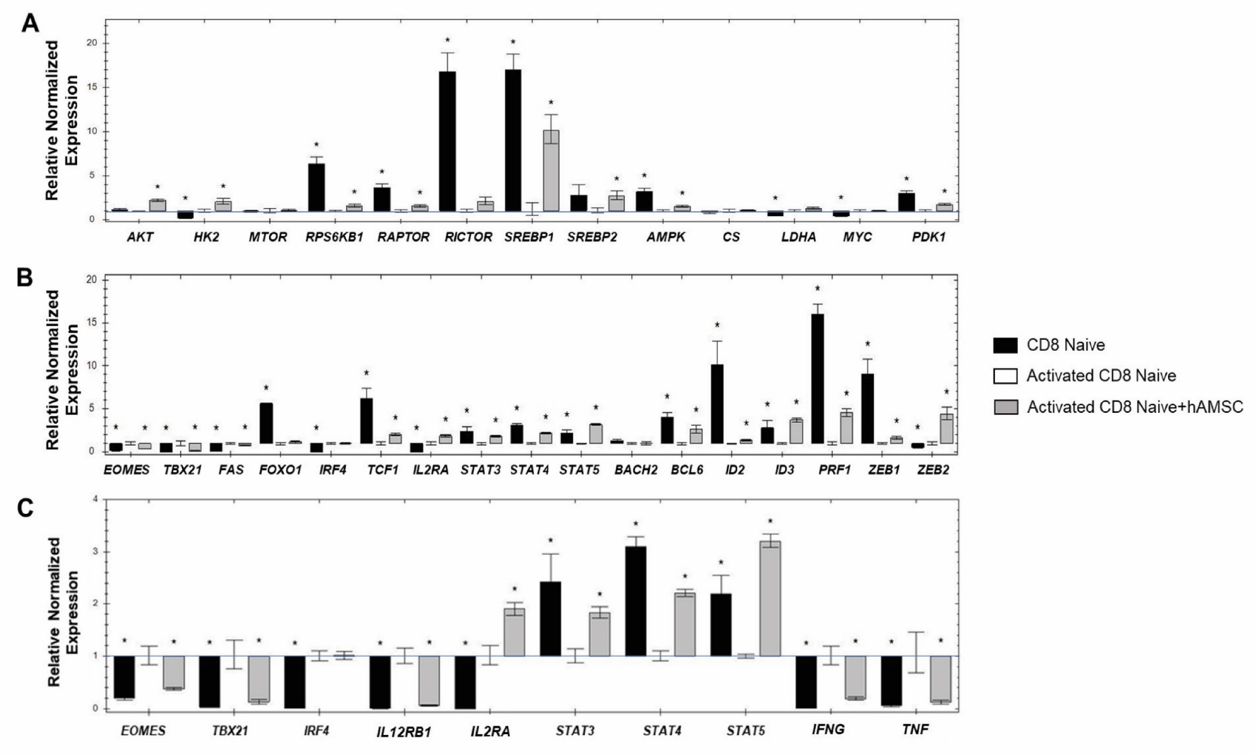

Figure S. 3. Gene expression analysis

Gene expression analysis for the genes presented in the hierarchical matrix in figure 5. A) Metabolic, B) Epigenetic and transcription factors and C) Naïve differentiation and commitment. Values are normalized versus the control condition represented by activated naïve CD8 T lymphocytes. Black bars: expression value of freshly isolated naïve CD8 T lymphocytes; gray bars: gene expression after co-culture with hAMSC. Data are represented as normalized fold change expression in comparison to the control condition represented by activated CD8 Naïve T lymphocytes and are reported as mean ± SEM. N = 3 independent experiments performed starting from 3 purified CD8 naïve donors and 5 different hAMSC preparation, (*p < 0.05).
